# Thermal Cyclotrimerization Bridges Polymer‐Filler Interfaces for High‐Performance CO_2_ Separation Membranes

**DOI:** 10.1002/advs.202520880

**Published:** 2026-01-18

**Authors:** Zhihong Lin, Kaifang Wang, Ziyi Yuan, Jiali Tang, Xiaozhen Liu, Liu Chen, Lu Shao, Xuezhong He

**Affiliations:** ^1^ Department of Chemical Engineering Guangdong Technion‐Israel Institute of Technology Shantou Guangdong China; ^2^ The Wolfson Department of Chemical Engineering Technion‐Israel Institute of Technology Haifa Israel; ^3^ State Key Laboratory of Urban Water Resource and Environment School of Chemistry and Chemical Engineering Harbin Institute of Technology Harbin China; ^4^ Guangdong Provincial Key Laboratory of Materials and Technologies for Energy Conversion Guangdong Technion‐Israel Institute of Technology Shantou Guangdong China

**Keywords:** CO_2_ separation, interfacial continuity, mixed matrix membranes, process simulation, thermal rearrangement

## Abstract

Mixed‐matrix membranes (MMMs) that combine polymers with porous fillers hold promise for scalable CO_2_ capture, but their performance is often limited by inadequate polymer–filler compatibility and suboptimal micropore structure. Here we demonstrate a thermal cyclotrimerization strategy that simultaneously tunes the microstructure of PIM‐1 and improves continuity at the MOFpolymer interface. Using MAF‐stu‐1 as a model filler, the resulting thermally rearranged MMMs achieve CO_2_ permeabilities above 10 000 barrer with a 2.5‐fold increase in CO_2_/N_2_ selectivity (from 18 to 46). Sorption measurements, spectroscopy, surface energy analysis, and molecular simulations reveal that cyclotrimerization introduces triazine units that both restrict polymer chain packing and create favorable interfacial interactions with MAF‐stu‐1, leading to enhanced pore accessibility and molecular discrimination. Importantly, these material‐level improvements translate into stronger process performance, reducing the specific CO_2_ capture cost by 43.9% compared to the untreated membrane. This work establishes thermal cyclotrimerization as a versatile strategy for engineering robust MMMs, linking interfacial chemistry to process‐level outcomes and advancing the development of practical membrane technologies for industrial carbon capture.

## Graphical abstract

Thermal cyclotrimerization of nitriles converts interfacial discontinuity in MAF‐stu‐1/PIM‐1 MMMs into a continuity‐enhanced junction. Triazine–Zn^2+^ coordination between TR‐PIM chains and MAF‐stu‐1, together with hydrogen bonding and π–π stacking, lowers interfacial tension and builds selective CO_2_ pathways, enabling > 10 000 barrer CO_2_ permeability, ∼2.5‐fold higher CO_2_/N_2_ selectivity, and ∼44% lower capture cost.

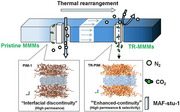

## 1 Introduction

Mixed‐matrix membranes (MMMs) represent a cutting‐edge breakthrough in membrane technology [1, 2], playing a pivotal role in tackling pressing industrial and environmental challenges [3, 4]. These advanced hybrid structures seamlessly combine the flexibility and processability of polymer matrices with high‐selectivity fillers, resulting in significant enhancements in selectivity and permeability. When it comes to CO_2_ separation, MMMs present an exceptional opportunity for effective carbon capture from flue gases. This is achieved through the selective sorption and diffusion of CO_2_ over N_2_, harnessing the strong affinity of the fillers for CO_2_ along with their molecular sieving properties. However, it is important to note that current MMMs still grapple with the inherent trade‐off between selectivity and gas permeability, often referred to as Robeson's upper bound [5]. This challenge has been a substantial barrier to their widespread implementation [6]. To unlock the full potential of MMMs, the strategic selection of fillers and the enhancement of phase integration with the polymer matrix are critical. This optimization will not only boost performance but also contribute to structural coherence relevant to membrane reliability.

Metal–organic frameworks (MOFs) are promising materials for CO_2_ capture due to their ultrahigh surface areas, intricately tunable pore structures, and strong affinity for CO_2_. They allow for precise optimization of adsorption properties [7, 8]. Certain MOFs exhibit high selectivity for CO_2_ over N_2_ under specific conditions, owing to their unique and diverse structural features, which include size‐selective pores and adaptable microenvironments that promote supramolecular interactions [9]. For example, Calgary Framework 20 (CALF‐20) [10] has been noted for its ability to adsorb CO_2_ at a higher capacity than N_2_ while maintaining selectivity in the presence of water. This selectivity is achieved through supramolecular interactions and physisorption driven by attractive dispersion forces. Reticular chemistry contributes to exceptional CO_2_ selectivity that can theoretically approach infinite discrimination [11, 12, 13]. Numerous MOFs have been incorporated into polymer matrices to fabricate MOF‐based MMMs via physical mixing, leading to significant advancements in CO_2_ separation technology [14, 15, 16, 17]. Nonetheless, filler‐polymer interfacial incompatibility and discontinuity remain a major bottleneck, limiting the full‐scale application of MOF‐based MMMs in CO_2_ capture. Semino et al. [18] revealed an interfacial gap of approximately 9 to 15 Å between the polymer of intrinsic microporosity‐1 (PIM‐1) matrix and ZIF‐8 fillers, indicative of poor interfacial continuity. This interfacial discontinuity highlights how poor filler‐polymer contact can hinder MOF accessibility and diminish separation performance, reinforcing the need for continuous interface design in MMMs [19]. To strengthen interfacial interactions, thereby improving interfacial compatibility and continuity, strategies such as MOF‐polymer cross‐linking [20, 21] and the introduction of additives [22, 23] have been reported. Moreover, molecular dynamics simulations have shown that controlling the nanostructure of the filler‐polymer interface can facilitate guided molecular transport. For example, the interfacial pore nanostructuring in AlFFIVE‐1‐Ni/PIM‐1 membrane [24] has been demonstrated to be an optimized, guided molecular pathway that minimizes entrance effects and accelerates transport and improves CO_2_ permeability and selectivity. Despite these advancements, achieving precise control over the MOF‐polymer interfacial microstructure requires advanced fabrication techniques, which increase the complexity of the membrane preparation process. Therefore, minimizing interfacial discontinuity through simpler methods, ideally without the use of additives, could enhance membrane performance and lead to a more cohesive and continuity‐enhanced interfacial structure.

Thermal treatment (rearrangement) emerges as a promising strategy to address these challenges [25]. Thermally driven structural rearrangements in polymer matrices can enhance chain adhesion to filler surfaces and foster the development of strong interfacial interactions [26, 27, 28, 29]. Different from other compatibility‐ or continuity‐enhancement strategies, thermal rearrangement offers the distinct advantage of preserving the intrinsic functionality of thermally stable MOF fillers. It avoids the risk of pore blockage commonly associated with additive incorporation and eliminates the need for specific reactive sites required in conventional filler‐polymer crosslinking. Furthermore, it enables mechanistic exploration of how interfacial chemistry governs gas transport. Yet, the limited multi‐level understanding of dynamic filler‐polymer interfaces hampers rational design and restricts insight into the underlying performance enhancements. Triazine‐forming thermal treatments of PIM‐1 have been successfully applied in thin‐film composite membranes, which already show strong promise for industrially relevant solvent resistance and CO_2_‐selective separations [30, 31], but triazine‐bridged MOF‐based MMMs have not yet been systematically investigated, even though such studies are essential to establish a mechanistic foundation for industrial implementation.

In this work, we focus on multi‐level interfacial chemistry and its crucial role in achieving continuity‐enhanced and high‐performance MOF‐based MMMs through polymer in situ thermal rearrangement. The selected polymer matrix, PIM‐1, is favored for its complementary properties and its emergence as a highly desirable membrane material for gas separation. After undergoing thermal rearrangement, the ─C≡N groups in PIM‐1 engage in a self‐crosslinking process, resulting in the formation of a triazine ring [32, 33]. This triazine ring, characterized by its three nitrogen atoms, promotes coordination or hydrogen bonding. Additionally, its inherent electron deficiency allows for a wide range of supramolecular interactions, such as π‐π stacking and σ‐bonding [34]. Nevertheless, to tackle the interfacial challenges, it is essential to use MOFs that exhibit robust thermal stability and well‐engineered properties to enhance compatibility with the polymer matrix during thermal rearrangement. Therefore, we consider MAF‐stu‐1 [35], an imidazole‐based framework with thermal stability up to 680°C, as a promising candidate for integration into the PIM‐1 matrix, allowing us to implement a targeted thermal rearrangement strategy. MAF‐stu‐1's periodically arranged binding pockets facilitate the precise recognition and capture of CO_2_ molecules, utilizing a mechanism known as shape complementarity. Unlike classic MOFs that rely on flexible binding, the rigid structural alignment of MAF‐stu‐1 ensures consistent CO_2_ adsorption efficiency with minimal energy consumption, making it an ideal filler for gas separation, particularly in MMMs.

We hypothesize that thermal rearrangement enables triazine‐mediated supramolecular interactions such as coordination with unsaturated zinc (Zn^2+^) [36], hydrogen bonding, and geometric π‐π stacking to form at the filler‐polymer interface. These cooperative, non‐covalent interactions are of particular interest for enhancing interfacial continuity. Most critically, this continuity‐enhanced interface is expected to facilitate CO_2_ transport into MAF‐stu‐1, leveraging shape complementarity to enhance separation performance in the resulting MMMs. To validate this hypothesis, a comprehensive investigation merging experimental works and theoretical simulations was undertaken to deepen our understanding of the effectiveness of MMMs in CO_2_/N_2_ separation. Moreover, the analysis of chemical bonding and molecular‐level interactions between MAF‐stu‐1 and PIM‐1 following thermal rearrangement was carried out using various techniques and simulations. Therefore, this integrated approach aims to clarify the role of interfacial chemistry in governing membrane performance, ultimately driving advancements in this field.

## 2 Results and Discussion

### 2.1 Preparation and Characterization of MAF‐stu‐1 and Pristine MMMs

**FIGURE 1:**
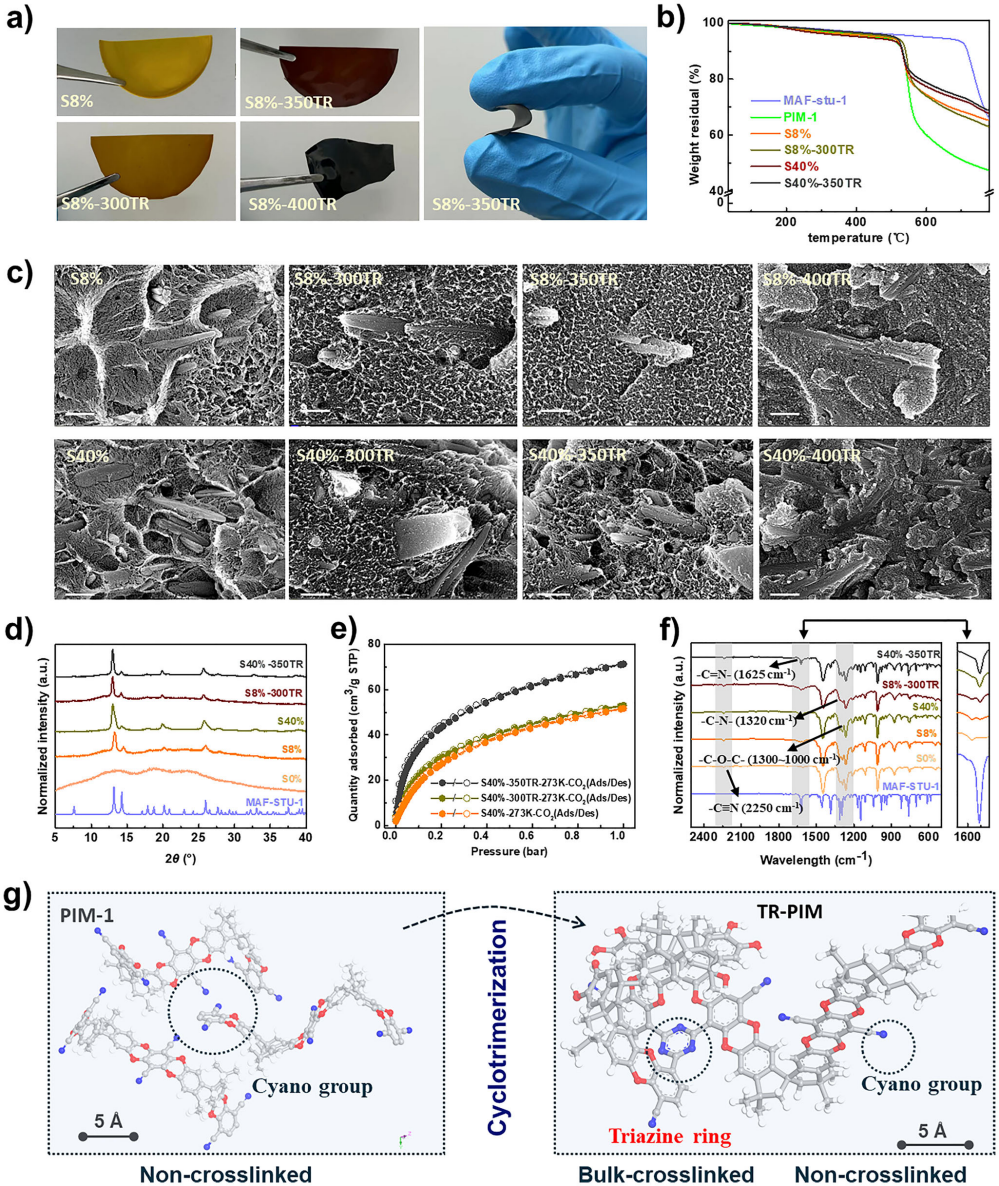
(a) Digital photographs of flexible S8% membranes before and after thermal rearrangement at 350°C and 400°C; (b) Thermogravimetric profiles of MAF‐stu‐1 and different membranes; (c) SEM cross‐sectional images (scale: 200 nm) of polymer and thermally‐treated membranes of S8% and S40%; (d) PXRD patterns of MAF‐stu‐1 and different membranes; (e) CO_2_ adsorption isotherms of S40% TR‐MMM; (f) FTIR spectra of different membranes and MAF‐stu‐1; (g) A schematic representation depicts the cyclotrimerization of PIM‐1 into TR‐PIM as a result of thermal rearrangement.

MAF‐stu‐1 [35] stands out for its remarkable ultrahigh thermal stability and exceptional shape complementarity for CO_2_. Powder X‐ray diffraction (PXRD) analysis confirmed the successful synthesis of MAF‐stu‐1, demonstrating high crystallinity and purity (refer to Figure S1). Notably, thermogravimetric analysis (TGA, Figure 1b) revealed its impressive thermal stability, capable of withstanding temperatures as high as 680°C—far surpassing the thermal tolerance of typical MOFs. A thorough analysis of CO_2_ physisorption at 273 K (see Figure S2) showcased an outstanding CO_2_ uptake capacity of 117 cm^3^/g, alongside a micropore volume of approximately 0.19 cm^3^/g and pore apertures ranging from 4 to 5 Å (see Figure S1). The distinct pore structure of MAF‐stu‐1 is meticulously engineered to optimally accommodate CO_2_ molecules, a feature described as CO_2_ shape complementarity. This design suggests a finely tuned configuration that significantly enhances the material's effectiveness in recognizing and capturing CO_2_. Furthermore, N_2_ and CO_2_ physisorption analysis at 298 K (see Figure S3) highlighted a significant difference in adsorption between CO_2_ and N_2_, pointing to the promising applicability of MAF‐stu‐1 in selective adsorption for enhancing membrane separation efficiency.

Self‐standing MAF‐stu‐1@PIM‐1 MMMs (see Figure 1a) were successfully prepared through a direct mixing method, achieving a high filler loading of up to 40 wt.%, surpassing the capabilities of most MOF‐based MMMs. Scanning electron microscopy (SEM) images reveal localized regions in which the rod‐like MAF‐stu‐1 fillers appear aligned parallel to the membrane surface within the PIM‐1 matrix (see Figure 1c). The closely matched densities of MAF‐stu‐1 (1.478 g/cm^3^) and the solvent chloroform (1.490 g/cm^3^) are critical in optimizing both the loading capacity and the uniform dispersion of the fillers [10]. This density matching facilitates homogeneous particle distribution within the PIM‐1 matrix and minimizes sedimentation or aggregation, contributing to improved membrane structural integrity and interfacial continuity to a certain extent. However, while signs of interfacial discontinuities between the PIM‐1 matrix and the MAF‐stu‐1 fillers are observable at an 8 wt.% filler loading, they become more pronounced at the higher loading of 40 wt.%. This observation underscores the challenge of limited filler‐polymer adhesion, which remains a critical barrier to fully realizing the performance potential of these advanced materials.

### 2.2 Preparations and Characterizations of Thermally Rearranged MMMs

To effectively design the thermal rearrangement protocol, we performed thermogravimetric analysis (TGA) on pristine PIM‐1 membranes, which revealed a significant thermal degradation onset at nearly 450°C, as illustrated in Figure 1b. Our subsequent analysis using Fourier Transform Infrared Spectroscopy (FTIR) demonstrated striking spectral changes in the PIM‐1 membranes subjected to thermal rearrangement, as shown in Figure S4. Correspondingly, a decrease in the characteristic ─C≡N stretching band at ∼2250 cm^−1^ was observed, accompanied by the emergence of a distinct peak at 1625 cm^−1^. This new peak corresponds to the ─C═N─ group, confirming triazine formation via in situ cyclotrimerization of ─C≡N groups in the PIM‐1 backbone. Concurrently, the disappearance of peaks between 1000 and 1300 cm^−1^ in 400°C‐treated PIM‐1 indicates cleavage of ─C─O─C─ bonds, signifying polymer backbone degradation. It should be noted that the observed discrepancies between the TGA and FTIR results may stem from differences in heating rates and soak times. Drawing from the comprehensive insights provided by the TGA and FTIR analyses, we reasonably established the thermal rearrangement protocol for MMMs at targeted temperatures of 300°C, 350°C, and 400°C, setting the stage for further investigation.

**FIGURE 2:**
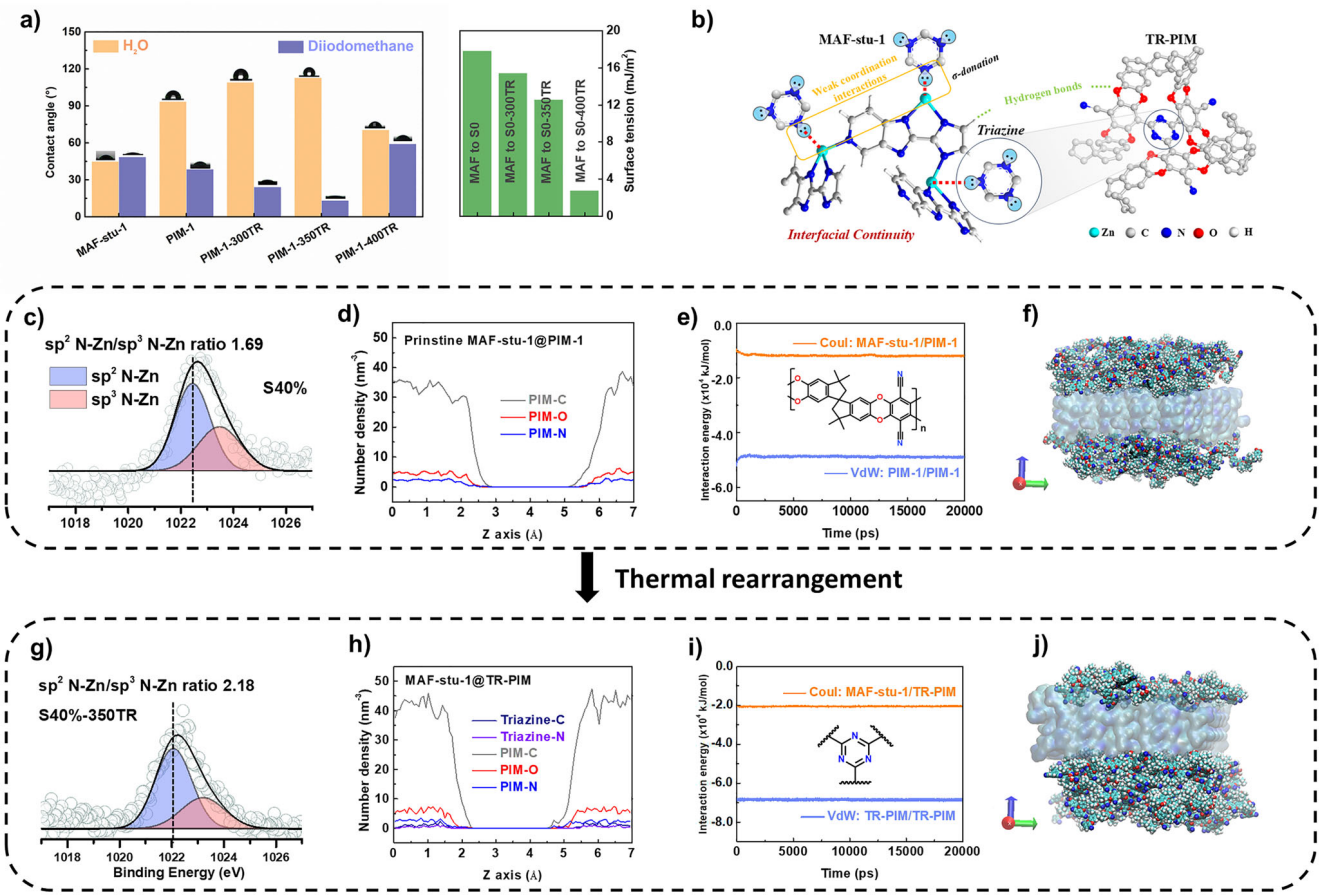
(a) Interfacial surface energy components calculated from contact angle measurements using the Owens–Wendt method. (b) Schematic illustration of the directional coordination between triazine nitrogen atoms and unsaturated Zn^2+^ sites in MAF‐stu‐1. (c, g) XPS analysis of the Zn 2p_3/2_ region, quantifying the ratio of sp^2^ N–Zn to sp^3^ N–Zn species in MAF‐stu‐1/PIM‐1 and MAF‐stu‐1/TR‐PIM systems, respectively. (d, h) Molecular dynamics–derived number density profiles of key atomic species at the filler–polymer interface. (e, i) Time‐dependent Coulombic and van der Waals interaction energies for the MAF‐stu‐1/PIM‐1 and MAF‐stu‐1/TR‐PIM systems. (f, j) Visual Molecular Dynamics (VMD) representations of the polymer–filler interface, where the polymer chains are rendered in CPK style with opaque surfaces, and the MAF‐stu‐1 is shown in a transparent QuickSurf model.

The synthesized MMMs underwent controlled thermal rearrangement at 300°C (denoted as Sx‐300TR), 350°C (Sx‐350TR), and 400°C (Sx‐400TR), The letter “x” indicates the loading percentage of MAF‐stu‐1: S8% for 8 wt.% loading and S40% for 40 wt.%, following the protocol outlined in Figure S5. A prolonged processing time of 48 h was employed to ensure maximum cyclotrimerization of the ─C≡N groups, which enhances interfacial adhesion between the two components within the MMMs. As the rearrangement temperature increased, the color of the thermally rearranged MMMs (TR‐MMMs) darkened. Membranes treated at 300°C and 350°C retained transparency, while those subjected to 400°C became opaque (see Figure 1a) due to partial degradation and carbonization of the PIM‐1 backbone. Notably, TR‐MMMs maintained good flexibility after treatment at 350°C; however, rearrangement at temperatures above 400°C resulted in brittle and fragile membranes, which will not be discussed further here. Moreover, thermal rearrangement prevents the development of discontinuous interface morphology, as confirmed by SEM cross‐sectional images (see Figure 1c), showing conformal polymer wrapping around MAF‐stu‐1 particles, and further supported by surface energy analysis, XPS, and MD simulations (see Figure 2 and Interfacial interaction mechanisms section). In comparison to the untreated membranes, the adhesion between MAF‐stu‐1 and PIM‐1 was significantly improved. At 400°C, TR‐MMMs also displayed visually polymer wrapping alongside significant changes in polymer morphology. However, this morphological transformation compromises the polymer structure, making it unsuitable for gas separation applications. Mechanical tests (see Figure S28) reveal that adding rigid MAF‐stu‐1 fillers transforms neat, relatively ductile PIM‐1 into a stiffer, more brittle composite by introducing stress concentrators, and that subsequent triazine‐forming thermal rearrangement further tightens and embrittles the glassy network. This becomes severe at 40 wt.% where a dense particle network and defect‐sensitive interfaces markedly reduce tensile strength and strain at break, whereas at 8 wt.% the membranes remain sufficiently extensible for practical handling.

PXRD analysis, illustrated in Figure 1d, clearly demonstrates that the MAF‐stu‐1 fillers retain their structural integrity within the polymer matrix, even after an extended period of thermal rearrangement. This remarkable stability ensures that the MOFs maintain their functionality in the TR‐MMMs. Furthermore, the TGA results reveal that the incorporation of MAF‐stu‐1 particles enhances the thermal stability of the PIM‐1 matrix, as evidenced in Figure 1b; Figure S6. Furthermore, all membranes exhibit a marked increase in residual weight at elevated thermal rearrangement temperatures and higher filler loadings. This observation is a strong indication of improved thermal stability due to the synergistic effects of thermal crosslinking and the presence of Zn^2+^ atoms within MAF‐stu‐1. Notably, a weight loss plateau emerges at approximately 700°C when the filler loading reaches 40 wt.%, showcasing the ultra‐thermal stability of MAF‐stu‐1.

Gas sorption experiments on TR‐MMMs were conducted for CO_2_ alongside N_2_ at different temperatures. MAF‐stu‐1 demonstrated minimal N_2_ adsorption. Therefore, only CO_2_ adsorption isotherms were analyzed in detail to elucidate the adsorption behavior. As illustrated in Figure S7, PIM‐1 achieved a CO_2_ adsorption capacity of 55 cm^3^/g at 1 bar and 273 K. In contrast, TR‐MMMs exhibited a slight reduction in CO_2_ adsorption capacity, likely associated with the loss of critical adsorption sites and pore accessibility during the heating process. As the MAF‐stu‐1 loading increased from 8% to 40% by weight, a marked decrease in CO_2_ adsorption capacity was observed in the pristine MMMs. This decline may arise from limited micropore accessibility due to insufficient filler‐polymer interfacial integration. However, TR‐MMMs displayed substantial enhancements in CO_2_ adsorption capacity at higher thermal rearrangement temperatures, particularly the S40%‐350TR membrane, which achieved a CO_2_ adsorption capacity exceeding 70 cm^3^/g, as depicted in Figure 1e. Furthermore, the CO_2_ adsorption isotherms of most TR‐MMMs primarily exhibit a type I(b) isotherm, characterized by a gradual slope at low relative pressures that level off the heterogeneous microporosity of PIM‐1. However, the CO_2_ adsorption behavior of S40%‐350TR reveals a shift toward a type I(a) isotherm, marked by a steep increase in adsorption at low relative pressures. This shift is supported by the Langmuir affinity constant (*b_i_*) presented in Table S3, where elevated *b_i_* values signal stronger CO_2_‐adsorbent interactions. Such evolution in isotherm shape and uptake suggests that thermal rearrangement facilitates the construction of continuous, gas‐accessible pathways across the filler‐polymer interface [19], thereby enabling fuller utilization of the MOF's intrinsic microporosity. These results suggest that thermal rearrangement fosters interfacial continuity, facilitating direct CO_2_ transport from the polymer matrix into the MOF filler.

The FTIR spectra in Figure 1f clearly illustrate the structural transformations of TR‐MMMs as a function of filler loading and thermal rearrangement temperature. The ─C═N─ vibration at 1625 cm^−1^, attributable to the imidazole ring from MAF‐stu‐1, intensifies with increased filler content. At higher thermal rearrangement temperatures, this enhancement is further amplified by an increase in the ─C─N─ peak near 1320 cm^−1^ [37], indicative of ongoing triazine ring formation in the MOF/PIM‐1 systems. Therefore, these observations support the conclusion that the presence of MAF‐stu‐1 does not exert a significant impact on the cyclotrimerization process. In tandem with FTIR, XPS spectra of carbon (C) and nitrogen (N) in the MMMs were analyzed in both pre‐ and post‐thermal rearrangement, along with those for MAF‐stu‐1 (refer to Figure 2b; Figure S23), to elucidate the critical transformation from ─C≡N to triazine rings. The observed decline in the characteristic C 1s peak at 286.2 eV and the N 1s peak at 399.5 eV after thermal rearrangement underscores the successful conversion of ─C≡N groups. Simultaneously, the emergence of new binding energies at 286.8 eV for C 1s, and 400.4 and 398.5 eV for N 1s, associated with >C═N─ and ═C─N─ groups [38], validates the formation of triazine rings. Taken together, the FTIR and XPS results (see Figure 1g) strongly support the transformation of pendant ─C≡N groups into triazine rings. The self‐crosslinking of PIM‐1 results in a reorganization of the polymer microstructure, which increases free volume due to the inefficient packing inherent in the bulky triazine rings [33]. Crucially, the presence of triazine rings at the interface between the PIM‐1 matrix and MAF‐stu‐1 mediates multiple interfacial interactions [39], thereby ensuring interfacial continuity and improving gas selectivity.

### 2.3 Interfacial Interaction Mechanisms

To achieve a comprehensive understanding and quantitative evaluation of the enhanced interfacial properties of TR‐MMMs, we employed the Owens–Wendt method to estimate surface energy (refer to Note S3). This approach effectively distinguishes between dispersive (non‐polar) interactions (γ*^d^*) and polar interactions (γ*^p^*) [40, 41, 42]. The interfacial surface energy of the polymer matrix and MAF‐stu‐1 was meticulously calculated using contact angle measurements with polar (water) and non‐polar (diiodomethane) liquids (Figure 2a; Figure S8). Specifically, the hydrophobicity of PIM‐1 membranes surged following thermal rearrangement at 300°C and 350°C, driven by the formation of hydrophobic triazine rings. In contrast, hydrophilicity spiked at 400°C due to the emergence of a graphene‐like surface resulting from partial carbonization [43].

Furthermore, the observed decrease in the diiodomethane contact angle, along with changes in both the dispersive *γ^d^* and polar *γ^p^* components (Table S1), clearly indicates a transition to a more hydrophobic and non‐polar surface with increased dispersive energy at 300°C and 350°C. Within the TR‐MMMs system, the potential specific triazine‐N/Zn coordination interactions were hypothesized to establish *γ^p^*. Conversely, the dispersive *γ^d^* rise is attributed to expanded π‐conjugation and transient π‐π stacking between aromatic domains of the polymer and MOF. The consistent enhancement of both *γ^d^* and *γ^p^* (Table S1) underscores the evolution of larger π‐conjugated domains and intensified coordination interaction within the TR‐MMM junction regions. Additionally, the gradual reduction in interfacial surface tension values (i.e., surface free energy difference) is a strong indication of a continuous and energetically favorable interface (17.78, 15.39, 12.54, and 2.74 mJ/m^2^). Initially, weak interactions uphold high surface tension, but the emergence of stronger interfacial interactions systematically lowers it. Therefore, thermal rearrangement fosters an energetically favorable interface that enhances filler‐polymer uniformity and continuity.

A clear downshift in Zn 2p_3/2_ binding energy (see Figure 2c,g) was observed upon thermal rearrangement, decreasing from 1022.42 eV in S40% (without thermal treatment) to 1022.03 eV in S40%‐350TR. Notably, this shift did not occur in the untreated membrane (see Figure S23), even at high MOF loading, indicating that thermal rearrangement is essential for activating new interfacial interactions. This subtle but definitive shift indicates an increase in local electron density around Zn atoms [44], which signifies the emergence of weak coordination interactions (see Figures S19 and S20). On the contrary, the remaining ─CN groups, due to their linear geometry and poor σ‐donor capacity, do not contribute significantly to binding energy shifting. Therefore, the observed binding energy shift arises from directional weak triazine‐N/Zn interactions, which induce local coordination field perturbations and result in partial Zn core shielding, thereby producing detectable spectral changes.

To further support this binding energy shift, the ratio of Zn─N═C─ (denoted as sp^2^ N─Zn) to Zn─N─C─ (sp^3^ N─Zn) species was analyzed via peak deconvolution (see Figure S23). For the low‐loading membrane S8%, the ratio decreased from 1.7 in pristine MAF‐stu‐1 to ∼1.21 after filler incorporation, owing to limited MOF surface exposure within the XPS probing depth (∼10 nm). Upon thermal rearrangement, this ratio increased to 1.92, indicating enhanced sp^2^‐type coordination. For the high‐loading membrane S40%, the ratio remained close to the pristine value at ∼1.69 prior to MAF‐stu‐1 but rose further to 2.18 after thermal rearrangement (see Figure 2c,g), consistent with increased triazine‐N/Zn coordination at higher MOF loadings. Although secondary to the binding energy shift, this coordination trend reinforces the role of triazine‐N in perturbation of the interfacial Zn environments. Although XPS probes only detect the near‐surface regions, the spectral features provide chemically specific evidence of the triazine‐N/Zn coordination. Combined with surface energy trends, these results indicate the formation of a continuous integrated interface.

Molecular dynamics (MD) simulations were further conducted to explore the spatial characteristics and dynamic behavior of these interactions [45, 46], and the results (refer to Note S4) reveal that thermal rearrangement induces molecular restructuring in TR‐MMMs. A MOF slab model (001) was constructed and integrated with either PIM‐1 or thermally rearranged PIM‐1 (TR‐PIM). The resulting simulation cells (see Figures S16 and S17) display clear differences in interfacial structure: the MAF‐stu‐1/PIM‐1 system exhibits prominent interfacial voids arising from the intrinsically contorted PIM‐1 backbone, whereas the TR‐PIM chains form a denser and more continuous interface. This improvement is attributed to the increased rigidity and steric bulk introduced by cyclotrimerization, which enhances physical adhesion and suppresses void formation.

In the MAF‐stu‐1/PIM‐1 system, MD‐derived number density profiles for various atoms reveal minimal interfacial mixing between the PIM‐1 matrix and the MAF‐stu‐1 fillers (as depicted in Figure 2d). This is evidenced by a steep decline in the density of PIM‐C atoms across a narrow interfacial region (z = 2–5 Å). Following thermal rearrangement, a distinct peak corresponding to triazine‐N emerges at the filler‐polymer interface (see Figure 2h), indicating increased proximity between triazine‐N and Zn centers—consistent with the Zn 2p_3/2_ binding energy shift observed in XPS (see Figure 2g). Following the mesoscopic insights from number density profiles, radial distribution functions (RDFs) enable atomistic resolution of local interactions, particularly between N and Zn in different systems. As shown in Figure S19, triazine‐N/Zn pairs exhibit a weak peak shifted toward higher r≈3.5 Å, reflecting imposed limitations on interfacial triazine cyclotrimerization. To assess interfacial contacts more directly, site‐specific partial RDFs averaged over trajectory frames were computed between individual Zn atoms and nearby triazine‐N atoms from selected polymer segments (see Figure S20). These RDFs exhibit sharper maxima at ∼3.2–3.8 Å with g(r) > 1.0, confirming the short‐range Zn‐N correlations at select interfacial sites and providing spatial support for the electronic perturbation reflected in the Zn 2p_3/2_ shift. These localized features, although not pervasive across the system, suggest that the geometry‐specific weak triazine‐N/Zn coordination does occur in the thermally rearranged configuration. Additionally, RDFs for Zn─O and Zn─C pairs (see Figure S21) reveal broader and more continuous interfacial contact, further supporting the formation of a more continuous interface with high adhesion. Further evidence for interfacial reinforcement arises from hydrogen bonding analysis. The distribution of PIM‐O (oxygen from the polymer backbone) is more diffused (see Figure 2h), highlighting retained flexibility in non‐crosslinked regions (see Figure 1g) and suggesting its role as a hydrogen bond acceptor at the interface. This interpretation is supported by hydrogen bond analysis (see Figure S22), which identifies PIM‐O as the primary acceptor and hydrogen atoms from the MOF ligands as dominant donors. The thermally rearranged system (MAF‐stu‐1@TR‐PIM) exhibits a substantially higher average number of hydrogen bonds (∼20) compared to the pristine part (∼8), along with broader temporal fluctuations. These results indicate greater exposure and favorable orientation of acceptor groups on TR‐PIM and a more dynamic H‐bonding network that can contribute to interfacial cohesion.

To gain a more quantitative and statistically meaningful understanding of interfacial cohesion, we analyzed the time‐dependent Coulombic and van der Waals interaction energies in the filler‐polymer interface. These global energetic descriptors offer system‐averaged insights with greater statistical significance, complementing the local coordination behavior revealed by number density profiles, RDFs, and hydrogen bond analyses. As shown in Figure 2e,i, the pristine MAF‐stu‐1/PIM‐1 system exhibits relatively weak interfacial interactions manifest as low Coulombic (Coul: ∼ −4.0×10^3^ kJ/mol) and van der Waals (vdW: ∼ −6.0×10^3^ kJ/mol) energies, highlighting a lack of robust chemical bonding and consequently, poor interfacial adhesion. Upon thermal rearrangement, the interfacial Coul energy drops to approximately −1.1 × 10^4^ kJ/mol (see Figure 2i), which reflects an enhancement in electrostatic attraction. This energetic shift correlates with the RDF‐identified triazine‐N/Zn proximity and the Zn 2p_3/2_ binding energy downshift observed in XPS, suggesting the formation of transient, coordination‐like interactions at the interface, which are possibly associated with σ‐donation from triazine‐N to Zn^2+^. In addition, the increased number of interfacial hydrogen bonds also contributes further to the strengthened Coul energy. In parallel, the van der Waals becomes more negative (approximately −8.0×10^3^ kJ/mol), indicating strengthened non‐covalent interactions (particularly those arising from TR‐PIM), which promote potential π‐π stacking with the aromatic domains of the MAF‐stu‐1 framework. It should be noted that TR‐PIM/TR‐PIM self‐interactions achieve outstanding vdW cohesion of approximately −5.5×10^4^ kJ/mol (see Figure S24), driven by dense π‐π stacking within the bulker phase of the TR‐PIM backbone.

In summary, XPS, MD simulations, and surface tension analysis jointly construct a multi‐scale framework for understanding interfacial evolution upon thermal rearrangement. XPS reveals Zn 2p_3/2_ binding energy shifts indicative of weak triazine‐N/Zn coordination. MD analyses capture enhanced Zn‐N proximity, interfacial hydrogen bonding, and π‐π stacking, supported by density profiles, RDFs, and energy decomposition. These molecular‐level changes are reflected macroscopically by increased polar (*γ^p^*) and dispersive (*γ^d^*) surface energies, signaling improved cohesion and continuity. Together, these complementary techniques trace a coherent trajectory from orbital‐level coordination to molecular interaction energetics and bulk physical response (see Figure 2b). Thermal rearrangement thereby enables the construction of chemically integrated (see Figure 2f,j), structurally robust materials that facilitate CO_2_ transport into the MOF phase, advancing thermal rearrangement it as a generalized design principle for high‐performance membranes.

### 2.4 Membrane Gas Separation Performances

**FIGURE 3:**
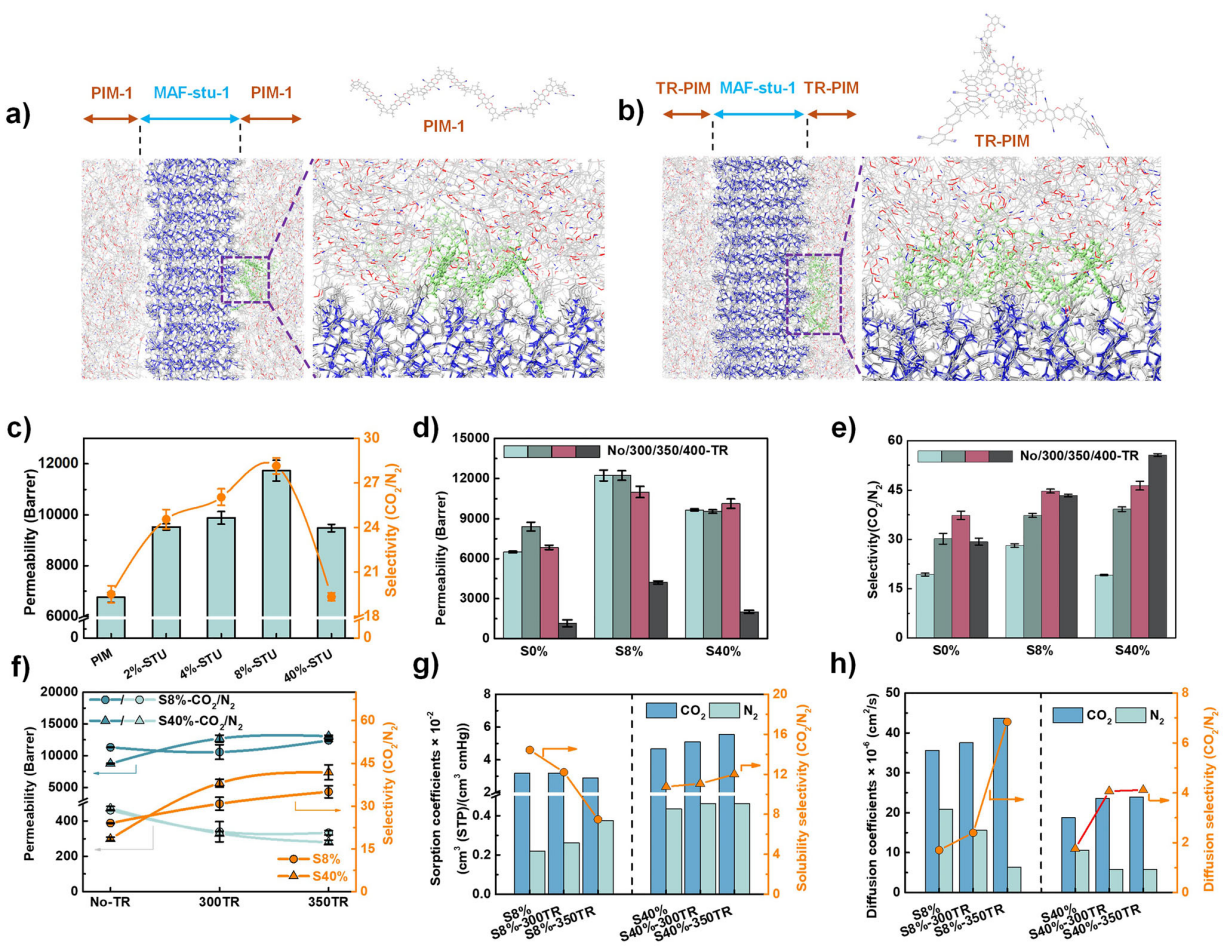
Snapshots of one of the MAF‐stu‐1/PIM‐1 (a) and MAF‐stu‐1/TR‐PIM (b) interface configurations. The following color code is used to represent the atoms in MAF‐stu‐1 and the polymer: Zn is shown in light blue, N in blue, C in gray, H in white, and O in red. The highlighted polymer chains are depicted in green. (c–e) The mixed‐gas (CO_2_/N_2_ = 10%/90% in volume) permeation performance, specifically CO_2_ permeability and CO_2_/N_2_ selectivity, as a function of the MAF‐stu‐1 loading and the thermal treatment temperature of the resulting membranes (labeled Sx, Sx‐300TR, Sx‐350TR). (f) The single gas permeation performances of different membranes were measured at a feed pressure of 0.5 bar. (g and h) Solubility and diffusivity coefficients calculated from CO_2_ adsorption isotherms at 298 K, which correlate with the observed ideal‐gas permeation results.

Figure 3a,b provides insights into the junction regions between the filler and the polymer, illustrating the way polymer chains intricately wrap around the (001) crystal faces of MAF‐stu‐1. In the MAF‐stu‐1/PIM‐1 system, visible interfacial discontinuity (as shown in Figure 3a) leads to significant CO_2_ and N_2_ buildup outside the sieving apertures of MAF‐stu‐1. This accumulation causes aperture blockage and limits filler performance. Conversely, molecular visualizations of the MAF‐stu‐1/TR‐PIM system (see Figure 3b; Figure S18) demonstrate polymer penetration and enhanced interfacial continuity. The bulkier TR‐PIM chains form a gear‐like interlocking structure, embedding into the MOF surface and establishing a continuous interface. This architecture facilitates CO_2_ access to MOF apertures, reduces diffusional resistance, and collectively contributes to the superior separation performance of TR‐MMMs.

**FIGURE 4:**
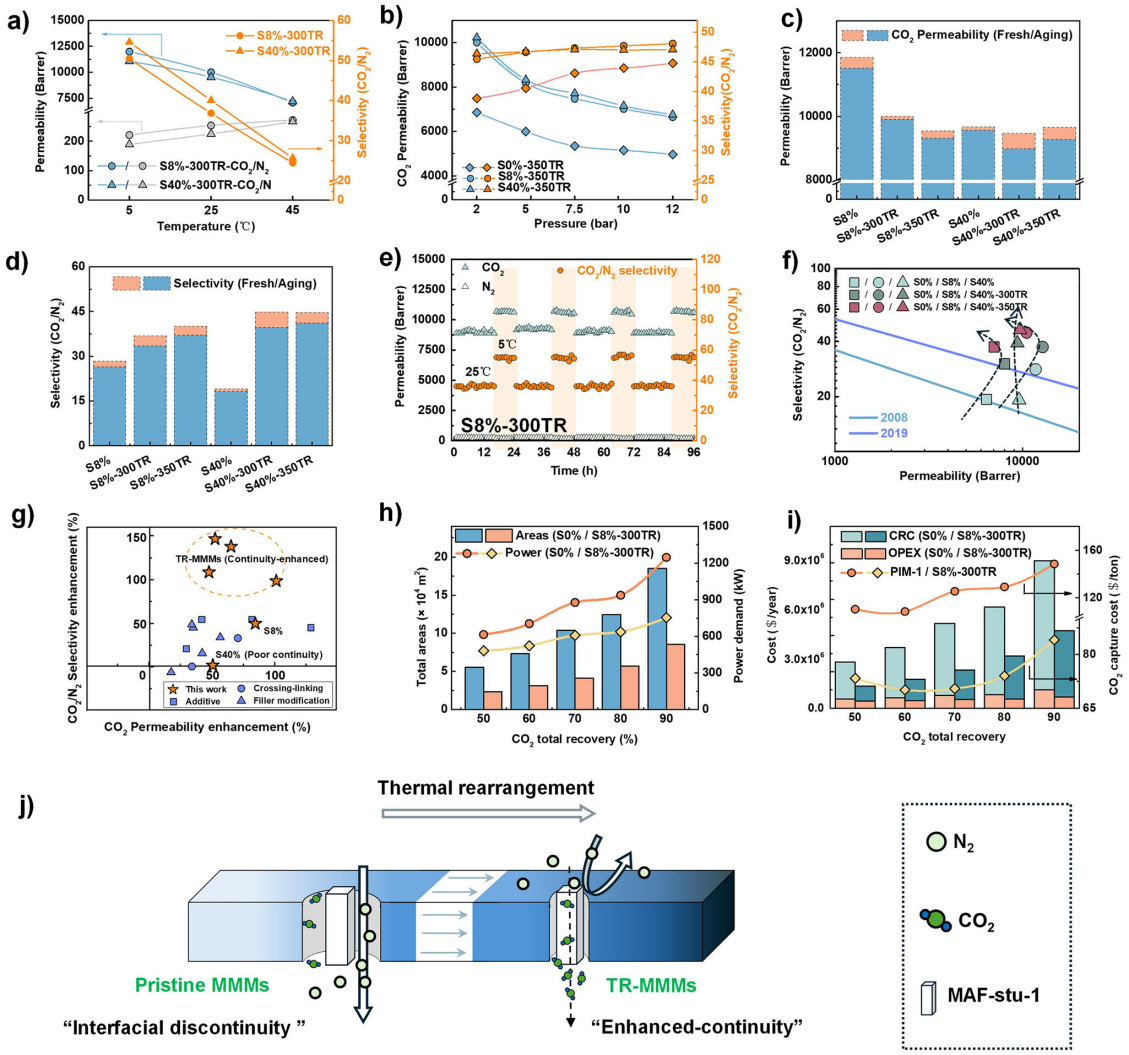
a) Temperature‐dependent mixed‐gas CO_2_/N_2_ (10%/90% in volume) permeation result on Sx‐300TR; (b) Pressure‐dependent mixed‐gas CO_2_/N_2_ permeation result on Sx‐300TR over the 2–12 bar range; (c, d) Separation performance of membranes before and after 21‐day ambient aging; (e) Long‐term operational stability of S8%‐300TR across a range of temperatures; (f) CO_2_/N_2_ separation performance compared to Robeson upper bounds (2008, 2019); (g) Enhancements in CO_2_/N_2_ selectivity and CO_2_ permeability compared to previously reported interfacially modified MMMs; (h) Process metrics at varying CO_2_ total recovery for PIM‐1 and S8%‐300TR, showing total membrane areas and instantaneous total power; (i) CRC (capital‐related cost), OPEX (operating expenditure) and specific CO_2_ capture cost across recoveries; (j) A schematic diagram reveals the interfacial morphologies evolution and the mechanisms driving gas separation of TR‐MMMs.

The CO_2_/N_2_ separation performance of the membranes, with MAF‐stu‐1 loading varying from 2 to 50 wt.%, was tested using a CO_2_/N_2_ mixture (10:90, v/v) at a feed pressure of 2 bar and a temperature of 25°C. The results are presented in Figure 3c and Figure S9. It was observed that the addition of MAF‐stu‐1 gradually improves both CO_2_ permeability and CO_2_/N_2_ selectivity. Notably, the MMM with an optimal loading of S8% surpasses the 2019 Robeson upper bound, as illustrated in Figure 4e. However, the membrane with a higher loading of S40% demonstrated a decrease in CO_2_/N_2_ selectivity, approaching that of the pristine PIM‐1 membrane. This high filler loading capability is particularly relevant for future process scaling and demonstrates the potential for constructing continuous filler‐–polymer networks at elevated loadings. Before examining the effects of thermal rearrangement in prepared MMMs, it is instructive to first consider its influence on the pristine polymer. Upon thermal treatment under an inert atmosphere, pure PIM‐1 membranes exhibited a clear enhancement in CO_2_/N_2_ selectivity, accompanied by a moderate reduction in permeability (Figure 3d,e). This trend suggests that thermally induced cyclotrimerization reorganizes the microporous network and promotes size‐selective diffusion. A similar phenomenon has been reported by Song. et al. [47] who attributed selectivity improvements in oxidatively crosslinked PIM‐1 to the narrowing of micropore bottlenecks. Although the underlying chemistries differ, both cases highlight the effectiveness of thermal rearrangement in tuning intrinsic transport properties. To explore this effect in greater depth, we investigated the influence of prolonged thermal treatment. It was found that extending the thermal rearrangement time to 48 h at 300 and 350°C had a minimal impact on CO_2_ permeability but significantly enhanced CO_2_/N_2_ selectivity, as shown in Figure 3d,e (along with Figures S10 and S11). The membrane S40%‐350TR demonstrates a 2.5‐fold improvement in selectivity compared to the untreated S40% membrane. In contrast, membranes subjected to brief thermal treatment (e.g., one hour) exhibited no significant changes in separation performance, as shown in Figure S12. These results underscore the importance of prolonged thermal processing for achieving as much cyclotrimerization as possible. Notably, TR‐MMMs with higher filler loadings exhibited enhanced selectivity, following the trend: S40%‐350TR > S8%‐350TR > S0%‐350TR. This trend underscores the critical role of MAF‐stu‐1, not only by contributing intrinsic shape selectivity, but more importantly as a performance‐driving component whose full functionality is unlocked through thermal rearrangement. As shown in Figure S13, thermal rearrangement at 400°C significantly reduced CO_2_ permeability across all TR‐MMMs due to increased collisions within the polymer backbone. However, the CO_2_/N_2_ selectivity of S40%‐400TR increased to 55. The combined effects of polymer backbone collisions and a maximally continuous filler‐polymer interface (see Figure 1e,g) lead to peak CO_2_/N_2_ selectivity.

Single gas permeation tests conducted at 0.5 bar and 25°C (detailed in Note S1) have illuminated the separation mechanism in TR‐MMMs, as demonstrated in Figure 3f. The performances obtained from single gas permeation testing closely mirror the trends observed in mixed‐gas permeation experiments, a phenomenon likely resulting from the negligible competitive adsorption in mixed‐gas conditions, attributable to the ultra‐low N_2_ adsorption. Leveraging gas sorption data at 25°C (see Figure S7) alongside single gas permeation results, the gas solubility and diffusivity coefficients (summarized in Tables S2 and S3) were calculated using the solution‐diffusion model (refer to Note S2) and visualized in Figure 3g,h. The S8% series revealed a modest decline in CO_2_ solubility and CO_2_/N_2_ solubility selectivity, primarily due to a reduction in specific adsorption sites following thermal treatment. In striking contrast, the S40% series exhibited a significant surge in CO_2_ solubility from 4.66×10^−2^ to 5.54×10^−2^ cm^3^(STP)/(cm^3^·cmHg), with a steadily increasing CO_2_/N_2_ solubility selectivity from 10.77 to 11.99. This improvement can be directly linked to the continuous filler‐polymer interface that enhances the reorientation of CO_2_ molecules, facilitating their interaction with MAF‐stu‐1 within the TR‐MMMs. For diffusivity, both series outperformed pristine PIM‐1 after thermal rearrangement, but the underlying mechanisms diverged at different filler loadings. In the S8%, the substantial increase in CO_2_ diffusion coefficients stems from thermal rearrangement, which expands the fractional free volume [32] of the polymer matrix, thereby enabling superior CO_2_ diffusion. Conversely, the S40% displayed lower CO_2_ diffusion coefficients due to the trapping of CO_2_ within MAF‐stu‐1, which limits gas diffusion [48, 49, 50]. Additionally, the CO_2_ diffusion selectivity saw significant boosts for both series post‐thermal treatment. The S8% series demonstrated selectivity enhancement, a result of the effective sieving action from the rearranged polymer backbone that substantially restricts N_2_ transport. In the S40% series, the moderate improvement in selectivity, from 1.76 to 4.11, stems from MOF‐induced confinement, again activated through interface continuity. Collectively, the diffusion‐dominated membrane S8%‐300TR exhibits a CO_2_ diffusivity of 43.70×10^−6^ cm^2^/s and achieves a high CO_2_ permeability of 12 000 barrer, while the solution‐dominated S40%‐350TR delivers a CO_2_ solubility of 5.54×10^−2^ cm^3^(STP)/(cm^3^·cmHg) and a high CO_2_/N_2_ selectivity of 46, which is 2.5‐fold higher compared to the untreated S40% membrane (18). These advancements highlight the potential of these membranes in optimizing gas separation processes.

Temperature‐dependent mixed‐gas CO_2_/N_2_ permeation tests for the S8% and S40% membrane series were rigorously conducted at 5, 25, and 45°C, with the compelling results illustrated in Figures 4a and Figure S14. At the lower temperature of 5°C, the membrane achieved a substantial enhancement in CO_2_/N_2_ selectivity, coupled with a substantial increase in CO_2_ permeability. In the S40% series, membranes exhibiting limited interfacial continuity (S40%) showed only modest improvements in CO_2_/N_2_ selectivity at lower temperatures. However, membranes with continuity‐enhanced interfaces, such as S40%‐300TR and S40%‐350TR, demonstrated substantial gains, with CO_2_/N_2_ selectivity rising from 40 to 54 and from 44 to 60, respectively. Furthermore, adsorption isotherms for CO_2_ and N_2_ were analyzed at 0°C and 25°C for both series (refer to Figure S7). At 0°C, CO_2_ adsorption capacity increased appreciably, while N_2_ adsorption remained relatively stable. Leveraging the solution‐diffusion mechanism, this increase in CO_2_ adsorption effectively compensates for the reduction in CO_2_ diffusivity at lower temperatures, resulting in enhanced CO_2_ permeability. Concurrently, the low solubility of N_2_, combined with diminished N_2_ diffusivity, led to a drop in N_2_ permeability. In conclusion, the CO_2_‐philic MAF‐stu‐1 promotes a CO_2_ solution‐dominated mechanism that measurably enhances CO_2_/N_2_ separation. Additionally, the continuity‐enhanced interface formed through thermal rearrangement optimizes CO_2_ solubility in MAF‐stu‐1, enabling striking membrane performance at low temperatures. This innovative approach positions these membranes as highly effective solutions for increased separation efficiency under a range of operating conditions.

Rigid glassy polymers like PIM‐1 exhibit susceptibility to plasticization, which manifests as a significant increase in permeability at higher pressures, particularly beyond a critical threshold known as the “plasticization pressure.” This phenomenon can lead to a drastic reduction in selectivity. To thoroughly evaluate the membrane's resistance to plasticization, we conducted pressure‐dependent mixed‐gas CO_2_/N_2_ permeation tests. The results, shown in Figure 4b and Figure S15, reveal a clear trend: CO_2_ permeability decreases consistently as pressure increases from 2 to 12 bar. This reduction stems from the non‐linear behavior of CO_2_ adsorption, attributed to the saturation of adsorption sites at lower pressures. Interestingly, the CO_2_/N_2_ selectivity actually increases, likely due to enhanced tortuosity of the polymer matrix, which is further promoted by membrane compression at elevated pressures. Moreover, the variation in CO_2_/N_2_ selectivity is in membranes treated at 300°C compared to those treated at 350°C, especially within the 2 to 7.5 bar range. This difference underscores the crucial role of membrane compression in facilitating the adhesion of polymer chains to the filler surface, while the treatment at 350°C results in a highly continuous interface. Importantly, we observe no signs of plasticization up to 12 bar, despite the documented plasticization pressure of PIM‐1 being 10 bar. This enhanced operational stability is attributed to the synergistic effects of MAF‐stu‐1 incorporation and interfacial continuity, which introduce additional triazine‐mediated stabilizing interactions.

Membranes engineered with enhanced anti‐aging performance and operational stability can extend system lifespan and reduce both replacement frequency and associated maintenance costs. Physical aging tests were performed to assess the stability of the prepared membranes (refers to Figure 4c,d). All membranes displayed resistance to aging over 21 days in ambient air, reflecting only minor reductions in CO_2_ permeability and CO_2_/N_2_ selectivity, except for the untreated S40% membrane. The slight performance decline can likely be attributed to moisture adsorption in the polymer matrix, temporarily hindering CO_2_ diffusion within the MAF‐stu‐1 fillers when exposed to air. In contrast, TR‐MMMs with higher filler loadings revealed a more significant performance drop, with an average selectivity loss of 9.6% for S40%‐300TR and S40%‐350TR compared to just 6.6% for the S8% series. However, the pristine S40% membrane with limited interfacial continuity mitigated the effects of de‐functionalized fillers on performance decline. Complementary to this, long‐term stability tests (see Figure 4e) reveal that the S8%‐300TR membrane maintains high separation performance over 96 h of continuous gas flow, even with temperature fluctuations between 5°C and 25°C across multiple cycles. Considerably, the membrane not only performs optimally at a lower feed gas temperature of 5°C but also continues to show resilience despite a slightly reduced performance at elevated temperatures of 25°C. This consistent performance reveals the critical role of a stabilized, continuous filler‐polymer interface reinforced by strong interfacial interactions in enhancing matrix rigidity, suppressing chain relaxation, and ultimately boosting membrane durability under diverse operational conditions.

The CO_2_/N_2_ separation performance of TR‐MMMs was further assessed in relation to the permeability‐selectivity trade‐off benchmarks, and the results are shown in Figure 4f and Table S4. Notably, the pristine S8% membrane surpasses the 2019 upper bound [51] for CO_2_/N_2_ separation, signifying the strong intrinsic separation ability of MAF‐stu‐1 embedding. Conversely, the pristine S40% membrane, exhibiting insufficient interfacial continuity that imposes resistance to selective gas transport, suffers a considerable decline in selectivity, leveling off to that of pristine PIM‐1, though with improved CO_2_ permeability. However, these interfacial limitations were significantly minimized across all TR‐MMMs, including those treated at 400°C, enabling their CO_2_/N_2_ separation performances to exceed the 2019 upper bound. PIM‐1 membranes display a comparable trend alignment with the S8% series under thermal treatment at low MOF loadings. Conversely, the S40% series with elevated MOF loadings exhibits markedly different behavior, highlighting the pivotal influence of MAF‐stu‐1. Such advancements enhance membrane efficiency but also position these MMMs as promising candidates for industrial‐scale carbon capture and separation applications.

The separation efficiency of CO_2_ and N_2_ using temperature‐responsive TR‐MMMs was compared to other interfacially modified MMMs utilizing the PIM‐1 matrix, as demonstrated in Figure 4g. Traditional methods for fabricating mixed matrix membranes, such as the incorporation of ionic liquids (ILs), surface functionalization of fillers, and covalent crosslinking between fillers and the polymer matrix, often lead to enhanced CO_2_ permeability but fall short in significantly improving CO_2_/N_2_ selectivity. In contrast, TR‐MMMs exhibit a boost in selectivity, driven by the synergistic combination of the MAF‐stu‐1 structure and thermally driven interfacial continuity. The unique CO_2_ shape complementarity of MAF‐stu‐1 empowers the matrix with a high density of active sites, thus facilitating superior CO_2_ recognition and selective adsorption. Moreover, the thermally induced interfacial adhesion effectively alleviates interfacial discontinuities, as illustrated in Figure 4j. These synergistic effects empower the MAF‐stu‐1 fillers to function as a highly effective selective sieve, underscoring the pivotal role of filler architecture and interfacial cohesion in enhancing CO_2_/N_2_ separation efficiency.

To convey material‐level improvements into plant‐relevant metrics, we quantified how permeance‐selectivity improvements result in required membrane areas (m^2^) and power demands (kW), and specific CO_2_ capture cost ($/ton). A two‐stage counter‐current configuration [4, 52, 53, 54] (see Figure S25) was implemented in UniSim R471 in which Stage‐1 controls overall CO_2_ recovery (%), Stage‐2 enforces a high CO_2_ purity of 95 mol%, and the Stage‐2 retentate is recycled to the Stage‐1 feed; operating conditions used a pressure ratio of 20 and the given membrane costs (50–55 $/m^2^) (see Note S5). Under this scheme, CO_2_ capture costs show a shallow U‐shape with a broad minimum at 60%–70% recovery, where further recovery no longer offsets rising total areas and power demands.

In detail, moving from PIM‐1 to MMMs (S8%, S40%) yields concurrent decreases in total membrane areas and power demands at certain recovery, lowering CO_2_ capture cost under the same membrane cost. Advancing TR‐MMMs (S8%‐300/350TR, S40%‐300/350TR) amplifies these trends that the S8% series chiefly lowers power demand without penalties in membrane areas, whereas the S40% series achieves a modest reduction in power at high recovery (see Figures S26 and S27). At 70% recovery, the diffusion‐dominated S8%‐300TR (higher permeance and selectivity) improves plant‐level metrics versus PIM‐1 (see Figure 4h,i), reducing total areas by 60.4% (4.12×10^4^ vs 1.04×10^5^ m^2^), power demands by 30.7% (608.10 vs 877.90 kW), and CO_2_ capture cost by 43.9% (70.50 vs 125.60 $/ton). These reductions reflect the combined permeance–selectivity improvements of S8%‐300TR, yielding simultaneous improvements in total areas, power demand, and CO_2_ capture cost. Relative to S8%‐300TR, the solution‐dominated S40%‐350TR (higher CO_2_ selectivity) bring higher total membrane areas demand while lowering power requirement as the 95 mol% CO_2_ purity constraint tightens; across 50%–90% recovery, power requirements is consistently lower by ∼2% at 50%–60% and ∼9% at 70%–90% (≈ 8–66 kW), indicating a clear power‐side advantage in the purity‐limited regime. In practice, the high‐selectivity membrane offers conserved operating capacity on the compression/sweep side when purity control dominates, whereas the high‐permeance membrane remains area‐efficient at moderate recovery, where shrinking total areas primarily drives CO_2_ capture cost (see Tables S5 and S6). Together, these results establish that a clean materials‐to‐process mapping of permeance governs footprint, selectivity governs power, and thermal rearrangement shifts both in directions that lower cost within the same operating envelope.

## 3 Conclusions

The PIM‐1‐based MMMs, featuring an impressive filler loading of over 40 wt.% have been expertly crafted to achieve excellent CO_2_/N_2_ separation performance. The filler, derived from the metal‐azolate frameworks (MAF‐stu‐1), boasts extraordinary thermal stability up to 680°C and outstanding CO_2_ adsorption capacity, driven by an effective mechanism known as shape complementarity. An important step forward in this research is the development of a cohesive interface between the filler and polymer, realized through a systematic thermal rearrangement strategy that simultaneously facilitates the formation of selective CO_2_‐permeable pathways. This interfacial transformation is supported by SEM evidence of polymer wrapping around the filler, enhanced CO_2_ physisorption, and quantitative interface analysis through contact angle experiments and MD simulations. Spectroscopic data from XPS and FTIR reveal that thermal cyclotrimerization partially converts pendant ─CN groups into triazine rings, which engage in directional, weak coordination with unsaturated Zn^2+^ sites on MAF‐stu‐1. Moreover, triazine‐mediated hydrogen bonding and π‐π stacking serve as additional interactions that stabilize the filler‐polymer interface. These cooperative supramolecular interactions, rather than a single dominant factor, collectively contribute to the observed reduction in interfacial surface tension (from 17.78 to 2.74 mJ/m^2^). MD simulations further confirm that these interactions promote a homogenized and continuity‐enhanced interface, effectively enabling CO_2_ transport across the filler‐polymer junction. The thermally rearranged MMMs (S40%‐350TR) exhibit a 2.5‐fold increase in CO_2_/N_2_ selectivity (from 18 to 46) while maintaining CO_2_ permeability above 10 000 barrer, outperforming membranes modified via conventional compatibility‐enhancing strategies. Within the techno‐economic framework, the minimum specific CO_2_ capture cost of 70.09 $/ton occurs at 60% recovery for S8%‐300TR, which shows a 43.9% reduction compared to the untreated membrane. This pioneering approach not only highlights the potential for creating continuity‐enhanced MMMs with a highly compatible filler‐polymer interface but also provides insights into the interfacial chemistry that dictates membrane structure and optimizes gas separation performance. By integrating material innovation with process‐relevant performance, this approach paves the way for practical, scalable solutions in post‐combustion carbon capture with mixed matrix membranes.

## 4 Methods

### 4.1 Materials

High‐purity analytical solvents were obtained from Xilong Scientific Co., Ltd. (Shantou, China), and other chemicals were purchased from Aladdin Chemical (Shanghai, China); all materials were used without further purification, including 5,5',6,6'‐tetrahydroxy‐3,3,3',3'‐tertamethyl‐1,1'‐spirobisindane(TTSBI), 2,3,5,6‐tetrafluorobenzene‐1,4‐dicarbonitrile (TFTPN), K_2_CO_3_, N,N‐dimethylformamide (DMF), trichloromethane (CHCl_3_), methanol (MeOH), ethanol, polyphosphoric acid, 3,4‐diaminopyridine, 1H‐imidazole‐2‐carboxylic acid, NaOH, aqueous ammonia, Zn(NO_3_)_2_·6H_2_O. We produced ultrapure water using an advanced reverse osmosis technique, guaranteeing the highest standards of purity. Additionally, we acquired a precisely formulated mixed gas comprising 10 vol% CO_2_ and 90 vol% N_2_ from a reputable gas company in Shantou, China, further enhancing the reliability of our experimental conditions.

### 4.2 Synthesis of PIM‐1 and MAF‐stu‐1

PIM‐1 was synthesized through polycondensation reaction, where 10.2 g (0.03 mol) of TTSBI, 6 g (0.03 mol) of TFTPN, and 8.3 g (0.06 mol) of dried K_2_CO_3_ were combined in 200 mL of DMF. This mixture was stirred under a nitrogen atmosphere at 65°C for 72 h. After this period, the mixture was allowed to cool to room temperature before being poured into 500 mL of pure water to precipitate PIM‐1. The solid was then filtered and purified in 200 mL of CHCl_3_. Following this, it was reprecipitated from MeOH and dried in a vacuum oven at 110°C overnight, ensuring the production of high‐purity PIM‐1 with a yield of 85%. Gel permeation chromatography (GPC, CHCl_3_, 30°C, polystyrene standards) gave a number‐average molecular weight *M*_n_ of ∼37500 g/mol, a weight‐average molecular weight *M*_w_ of ∼80800 g/mol, and a dispersity *Đ* of ∼2.2.

Polyphosphoric acid (10 mL) was carefully added to a flask containing 2.18 g 3,4‐diaminopyridine (0.02 mol) and 2.24 g 1H‐imidazole‐2‐carboxylic acid (0.02 mol) for the synthesis of the ligand of MAF‐stu‐1 (called H_2_imPim). This mixture was then heated at 180°C for 6 h. After cooling to room temperature, it was washed with a 5 m NaOH solution to achieve neutralization. The resulting product was collected, followed by washes with water and acetone, and vacuum‐dried at 50°C overnight. The yield of H_2_imPim obtained was 90%. 30 mL of ultrapure water and 50 mL of ethanol were combined in a flask with 1.1106 g H_2_imPim (6 mmol). To this mixture, aqueous ammonia (25%) was gradually added under continuous ultrasound, ensuring the complete dissolution of H_2_imPim. Next, 20 mL of ultrapure water containing 1.78 g Zn(NO_3_)_2_·6H_2_O (6 mmol) was introduced, and the mixture was stirred at room temperature for 5 h, then filtered and washed with ethanol. After drying at 50°C, white microcrystalline powder was obtained as the final product, yielding 80% (based on H_2_imPim).

### 4.3 Preparation of MAF‐stu‐1@PIM‐1 MMMs (Sx)

To prepare MMMs, various MAF‐stu‐1 particles were dispersed in 10 g of CHCl_3_ and sonicated to prevent aggregation. Next, 800 mg (8 wt.%) of PIM‐1 powder was introduced into the MAF‐stu‐1 suspension. After sonication to remove air bubbles, we poured the resulting MAF‐stu‐1@PIM‐1 casting solution onto a PTFE watch glass. This setup was placed in a flat‐bottomed glass container to facilitate solvent evaporation at ambient temperature, over three days. Membranes were then automatically stripped from the PTFE substrate and were immersed in methanol for an overnight soak, followed by drying in a convection oven at 110°C for an additional overnight period. A series of MMMs labeled MAF‐stu‐1@PIM‐1 were systematically prepared, each sample as Sx, where ′x″ indicates the weight percentage of MAF‐stu‐1 in relation to the total of MAF‐stu‐1 and PIM‐1. The loading percentages spanned from 2 wt.% (S2%), 4 wt.% (S4%), 8 wt.% (S8%) … to a high 40 wt.% (S40%). The fabricated MMMs displayed an increase in thickness, ranging from approximately 100 µm to 160 µm, correlating with the increasing filler loading.

### 4.4 Thermal Rearrangement of MAF‐stu‐1@PIM‐1 (Sx‐300/350/400TR)

The MAF‐stu‐1@PIM‐1 precursors were subjected to a well‐controlled thermal rearrangement process within a three‐zone furnace, utilizing an inert argon (Ar) atmosphere and a constant flow rate of 300 cm^3^/min. This procedure was executed in three distinct phases: (1) Gradually heating the material from room temperature to 300, 350, and 400°C at a precise ramp rate of 5°C/min; (2) Sustaining the target thermal rearrangement temperature for 48 h; and (3) Natural cooling to room temperature. This rigorous approach resulted in the successful production of a series of MAF‐stu‐1@PIM‐1 samples, designated as Sx‐300/350/400TR, where “x” signifies the specific MAF‐stu‐1 loading.

### 4.5 Instruments and Characterizations

Powder X‐ray diffraction (PXRD) data were collected using a Rigaku MiniFlex 600 diffractometer, utilizing Cu Kα radiation. The diffraction patterns were scanned over a range of 4–50° (2θ) at a rapid speed of 10 °/min, with a fine step width of 0.02°. For thermogravimetric analysis (TGA), a thermogravimetric analyzers‐TA Instruments TGA‐55 was employed under a N_2_ atmosphere, with a controlled heating rate of 10°C/min up to 800°C, ensuring accurate thermal profiling. X‐ray photoelectron spectroscopy (XPS) analysis was conducted with an AMICUS X‐ray photoelectron spectrometer, providing insights into the elemental composition and chemical states of the materials. The gas adsorption capacities of MAF‐stu‐1, PIM‐1, and TR‐MMMs were obtained from N_2_ adsorption isotherms at 298 K and CO_2_ adsorption isotherms at 273 and 298 K measured on a Micromeritics ASAP 2020 system and were used to quantify gas uptakes and CO_2_ affinity. To assess surface properties, contact angle measurements for the thermally treated MAF‐stu‐1 and PIM‐1 were carried out with a high‐precision drop shape analyzer (Kruss Scientific, Germany). Furthermore, Fourier‐transform infrared spectroscopy (FTIR) analysis was performed on MAF‐stu‐1, PIM‐1, and TR‐MMMs using the reliable Nicolet 460 equipment, yielding valuable spectral data. Lastly, the structural characteristics, surface, and cross‐sectional images of MAF‐stu‐1, PIM‐1, and TR‐MMMs were captured using a ZEISS GeminiSEM 450 field emission scanning electron microscope (SEM) operated at an accelerating voltage of 10 kV.

### 4.6 Mixed‐Gas Permeation Testing

Mixed‐gas permeation of CO_2_/N_2_ (10%/90% in volume) was evaluated using a constant‐pressure, variable‐volume method. The prepared MMMs were installed in a plate‐and‐frame module with an effective area of 0.785 cm^2^. Both the feed gas and module temperatures were precisely maintained at 5, 25, and 45°C using a heat tracing system. A pressure differential between 2 and 12 bar was controlled by two back‐pressure controllers (BPC 1 and BPC 2, Alicat Scientific, USA), positioned at the retentate and permeate sides. The gas mixture (CO_2_/N_2_) was fed into the module at 300 NmL/min via a mass flow controller (MFC1, Bronkhorst), while simultaneously a sweep gas (Ar) was introduced at 20 NmL/min (MFC2, Bronkhorst). After a stabilization period of at least two hours, the permeate flow was recorded with a mass flow meter (MFM, Bronkhorst) and its composition was analyzed by a gas chromatograph (Shimadzu GC‐2014, Shimadzu, Kyoto, Japan). This setup was used to evaluate gas separation performance.

Based on the experimental data, gas permeability (barrer) for the mixed gas permeation was calculated based on Equation (1):

(1) $$P_{i}=\frac{lQ_{p}y_{i}}{A\left(x_{i}p_{f}-y_{i}p_{p}\right)}$$

where, *x_i_* is the mole fraction of compound *i* in the feed gas and *y_i_* is the mole fraction of *i* in the permeate gas, *l* is the thickness of membrane (cm), A is the membrane effective area (cm^2^), *p*_f_ is the feed pressure (cmHg), *p_p_* is the permeate pressure (cmHg) and *Q_p_* is the permeate gas flow rate (NmL/min).

The mixed gas selectivity (S_*ij*_) was calculated by Equation (2),

(2) $$S_{ij}=\frac{y_{i}/y_{j}}{x_{i}/x_{j}}$$

### 4.7 Statistical Analysis

Gas permeation measurements were performed on three independently cast membranes for each formulation (*n* = 3), and the reported values represent the mean ± standard deviation. All other characterization methods (e.g., TGA, FTIR, XPS, SEM/TEM, PXRD, gas sorption) were conducted as single measurements (*n* = 1), and representative datasets are shown. No formal hypothesis‐testing procedures were applied. Data processing and graphing were carried out using Origin and Microsoft Excel.

## Author Contributions

Z.L. performed in formal analysis, methodology, investigation, data curation, writing – original draft, visualization. Z.Y., K.W., X.L., and L.C. performed in investigation, writing – review, and editing. J.T. and L.S. performed in writing – review and editing. X.H. performed in conceptualization, supervision, writing – review and editing, funding acquisition, and project administration. All authors have read and agreed to the published version of the manuscript.

## Conflicts of Interest

The authors declare no conflicts of interest.

## Supporting information




**Supporting File**: advs73808‐sup‐0001‐SuppMat.docx.

## Data Availability

The data that support the findings of this study are available from the corresponding author upon reasonable request.
