# Supplementary material for: Thermal Cyclotrimerization Bridges Polymer‐Filler Interfaces for High‐Performance CO2 Separation Membranes
Source: Adv Sci (Weinh). 2026 Jan 18;13(17):e20880. doi: 10.1002/advs.202520880 (PMC13042685; doi:10.1002/advs.202520880)
Supplement: Supplementary file 1 — Supporting File: advs73808‐sup‐0001‐SuppMat.docx. [file ADVS-13-e20880-s001.docx]

**Supporting Information (SI)**

Thermal Cyclotrimerization Bridges Polymer-Filler Interfaces for High-Performance CO_2_ Separation Membranes

Zhihong Lin^1,2^, Kaifang Wang^1,2^, Ziyi Yuan^1,2^, Jiali Tang^1^, Xiaozhen Liu^1,2^, Liu Chen^1^, Lu Shao^3^, Xuezhong He^1,2,4,*^

^1^Department of Chemical Engineering, Guangdong Technion-Israel Institute of Technology, 241 Daxue Road, Shantou, Guangdong, 515063, China

^2^The Wolfson Department of Chemical Engineering, Technion-Israel Institute of Technology, Haifa 32000, Israel

^3^State Key Laboratory of Urban Water Resource and Environment, School of Chemistry and Chemical Engineering, Harbin Institute of Technology, Harbin, 150001, China.

^4^Guangdong Provincial Key Laboratory of Materials and Technologies for Energy Conversion, Guangdong Technion-Israel Institute of Technology, Shantou, Guangdong 515063, China

*: Corresponding authors: xuezhong.he@technion.ac.il (X. He)

**Contents**

[Supplementary Note 1. Single gas permeation testing 5](#_Toc209605486)

[Supplementary Note 2. Calculation of diffusion coefficient (D) and solubility coefficient (S) 5](#_Toc209605487)

[Supplementary Note 3. Calculation of interfacial surface tension 6](#_Toc209605488)

[Supplementary Note 4. Molecular dynamic simulations 6](#_Toc209605489)

[Supplementary Note 5. Process simulation and techno-economic analysis for the two-stage membrane cascade (post-combustion flue gas CO_2_ capture) 9](#_Toc209605490)

[Supplementary Figure 1. PXRD patterns and crystal structure of MAF-stu-1 12](#_Toc209605491)

[Supplementary Figure 2. CO_2_ adsorption isotherm under 273 K and the pore size distribution, based on the DFT model. 12](#_Toc209605492)

[Supplementary Figure 3. N_2_ adsorption isotherm under 298 K 13](#_Toc209605493)

[Supplementary Figure 4. FTIR measurement on PIM-1 thermally treated under 300, 350 and 400 °C. 14](#_Toc209605494)

[Supplementary Figure 5. Thermal rearrangement protocol under 300, 350 and 400 °C. 15](#_Toc209605495)

[Supplementary Figure 6. TGA analysis on pristine PIM-1 and MMMs pre and post-thermal rearrangement at different temperatures. 16](#_Toc209605496)

[Supplementary Figure 7. CO_2_ and N_2_ adsorption isotherm under different temperatures. 17](#_Toc209605497)

[Supplementary Figure 8. Contact angle measurements on MAF-stu-1 and PIM-1 polymer thermally treated under different temperatures by using water and diiodomethane. 18](#_Toc209605498)

[Supplementary Figure 9. Mixed gas separation performance of MAF-stu-1@PIM-1 MMMs at different loading amounts. 19](#_Toc209605499)

[Supplementary Figure 10. Mixed gas separation performance of MAF-stu-1@PIM-1 MMMs thermally treated under 300 °C for 48 hours at different loading amounts. 19](#_Toc209605500)

[Supplementary Figure 11. Mixed gas separation performance of MAF-stu-1@PIM-1 MMMs thermally treated under 350 °C for 48 hours at different loading amounts. 20](#_Toc209605501)

[Supplementary Figure 12. Mixed gas separation performance of MAF-stu-1@PIM-1 MMMs thermally treated under 300 and 400 °C for 1 hour at different loading amounts. 21](#_Toc209605502)

[Supplementary Figure 13. Mixed gas separation performance of MAF-stu-1@PIM-1 MMMs thermally treated under 400 °C for 48 hours at different loading amounts. 22](#_Toc209605503)

[Supplementary Figure 14. Temperature-dependent mixed-gas CO_2_/N_2_ permeation results on Sx and Sx-350TR membranes. 23](#_Toc209605504)

[Supplementary Figure 15. Pressure-dependent mixed-gas CO_2_/N_2_ permeation results on Sx-300TR and Sx-400TR membranes. 24](#_Toc209605505)

[Supplementary Figure 16. Snapshots of the MAF-stu-1/PIM-1 and MAF-stu-1/TR-PIM interface configurations. 25](#_Toc209605506)

[Supplementary Figure 17. Snapshots of the MAF-stu-1/PIM-1 and MAF-stu-1/TR-PIM interface voids. 26](#_Toc209605507)

[Supplementary Figure 18. Snapshots of structural configurations of MAF-stu-1@TR-PIM and MAF-stu-1@PIM-1 obtained from molecular dynamics simulations. 27](#_Toc209605508)

[Supplementary Figure 19. Radial distribution functions g(r) between all Zn²⁺ sites in MAF-stu-1 and N atoms from PIM-1 and TR-PIM. 28](#_Toc209605509)

[Supplementary Figure 20. Radial distribution functions g(r) between individual Zn^2+^ sites in MAF-stu-1 and neighboring triazine nitrogen atoms from two distinct polymer segments in the TR-PIM matrix. 29](#_Toc209605510)

[Supplementary Figure 21. RDF between Zn²⁺ sites in MAF-stu-1 and (top) C atoms and (bottom) O atoms from the surrounding polymer segments. 30](#_Toc209605511)

[Supplementary Figure 22. Time evolution of the number of hydrogen bonds formed between donor N-H groups on MAF-stu-1 and acceptor O atoms on the TR-PIM polymer backbone. 31](#_Toc209605512)

[Supplementary Figure 23. Cyclotrimerization of the -C≡N groups following thermal rearrangement, demonstrated through X-ray photoelectron spectroscopy (XPS) analysis of the Zn 2p, C 1s and N 1s spectra. 32](#_Toc209605513)

[Supplementary Figure 24. Time-dependent Coulombic and van der Waals interaction energies for the MAF-stu-1/PIM-1, MAF-stu-1/TR-PIM, PIM-1/PIM-1 and TR-PIM/TR-PIM systems. 33](#_Toc209605514)

[Supplementary Figure 25. Two-stage membrane cascade with recycle and vacuum-assisted sweep, used for post-combustion CO_2_ capture process simulations. 34](#_Toc209605515)

[Supplementary Figure 26. The sensitivity analysis of CO_2_ recovery on stage-wise and total membrane area, and power demand for PIM-1, MMMs, and TR-MMMs. 35](#_Toc209605516)

[Supplementary Figure 27. The sensitivity analysis of CO_2_ recovery on OPEX, capital cost, and annual CO_2_ captured, and specific CO_2_ capture cost for PIM-1, MMMs, and TR-MMMs. 36](#_Toc209605517)

[Supplementary Table 1 Contact angle measurement and surface tension analysis 37](#_Toc209605518)

[Supplementary Table 2 The diffusivity coefficient, solubility coefficient and their selectivities for different membranes at 0.5 bar. 38](#_Toc209605519)

[Supplementary Table 3 Sorption parameters of different membranes, obtained by the model fitting of the CO_2_ and N_2_ sorption isotherms. 39](#_Toc209605520)

[Supplementary Table 4. Comparisons of TR-MMMs performances on CO_2_/N_2_ selectivity and CO_2_ permeability with previously reported interfacial modified MMMs. 40](#_Toc209605521)

[Supplementary Table 5. Process simulation results of different membranes: required membrane area, cost, and compressor power demand under varying CO_2_ recoveries, serving as the basis for cost and performance evaluation. 41](#_Toc209605522)

[Supplementary Table 6. Techno-economic analysis of membrane processes: operating cost, capital cost, annual CO_2_ captured, and capture cost at different recoveries. 43](#_Toc209605523)

# Supplementary Note 1. Single gas permeation testing

Single gas permeation tests for CO_2_ and N_2_ were carried out using the highly efficient constant volume and variable pressure method within a low-pressure membrane permeation system. The prepared MMMs were expertly mounted in a plate-and-frame module, showcasing an effective membrane area of 0.785 cm². To ensure precise conditions, the feed gas temperature was rigorously maintained at 25 °C, while a pressure difference of 0.5 bar was applied across the membrane. Leveraging the gathered experimental data, the gas permeability for ideal gas permeation was accurately calculated using equation (S1), demonstrating the effectiveness of our approach and the reliability of the results.

$P_{i}= \frac{VL}{A}\frac{T_{0}}{T{P_{0}P}_{f}}\frac{dp}{dt}$ (S1)

where *Pi* is the gas permeability, expressed as a function of various system parameters. The term *VL/A* accounts for the geometric properties of the system, with *V* (cm^3^) being the volume of the permeated gas, *L* (cm) and *A* (cm^2^) the membrane thickness and effective area. The ratio *T*_0_*/P*_f_*P*_0_*T* adjusts the permeability measurement to standard temperature (*T*_0_: 273.15 K) and pressure (*P*_0_: 76 cmHg), while factoring in operating temperature (*T*) and final pressure (*P*_f_). Finally, *d*p*/d*t represents the rate of pressure (cmHg) change over time (s).

The ideal-gas selectivity ($\alpha_{ij})$ was calculated by equation (S2),

$\alpha_{ij}=\frac{P_{i}}{P_{j}}$ (S2)

# Supplementary Note 2. Calculation of diffusion coefficient (D) and solubility coefficient (S)

Gas adsorption experiments were conducted at 25 °C under pressures ranging from 0 to 1 bar. The adsorption behavior was analyzed using the Langmuir isotherm model, described by equation (S3):

$C_{i}=\frac{C_{i}^{'}b_{i}p_{i}}{1+b_{i}p_{i}+b_{j}p_{j}}$ (S3)

where *C_i_* is the equilibrium adsorption capacity of gases *i* (cm^3^(STP)/cm^3^); *C’_i_* denotes the saturated Langmuir adsorption capacity (cm^3^(STP)/cm^3^); The Langmuir affinity constant (1/cmHg) of gases *i* and *j* are denoted by *b_i_* and *b_j_*, respectively; And *p_i_* and *p_j_* represent the partial pressure (cmHg) of gases *i* and *j*, respectively. The solubility coefficients (*S_i_*) in a mixture were calculated by applying a Langmuir binary adsorption model, as specified by Equation (S4).

$S_{i}=\frac{C_{i}}{p_{i}}=\frac{{C'}_{i}b_{i}}{1+b_{i}p_{i}+b_{j}p_{j}}$ (S4)

Thus, *D_i_* can be calculated by evaluating the ratio of gas permeability to solubility coefficient, as stated in equation (S5)

$D_{i}=\frac{P_{i}}{S_{i}}$ (S5)

# Supplementary Note 3. Calculation of interfacial surface tension

The components of solid-surface free energy can be yielded with the contact angles with deionized water and diiodomethane through equation (S6).

$cos\theta=-1+\frac{2\sqrt{\gamma_{s}^{d}\gamma_{l}^{d}}}{\gamma_{l}}+\frac{2\sqrt{\gamma_{s}^{p}\gamma_{l}^{p}}}{\gamma_{l}}$ (S6)

where *θ* is the contact angle (°) with DI water or diiodomethane; $\gamma^{d}$ and $\gamma^{p}$ are the dispersion component and polar dispersion of the total surface free energy (mJ/m^2^), while the subscript characters “*s*” and “*l*” represent solid and liquid phases, respectively.

With the obtained data of the tested materials, the interfacial tension ($\gamma_{ij}$) between two phases (i and j) can be calculated by equation (S7).

$\gamma_{ij}={(\alpha_{i}-\alpha_{j})}^{2}+{(\beta_{i}-\beta_{j})}^{2}+\Delta_{ij}$ (S7)

where $\alpha_{s}=\sqrt{\gamma_{s}^{d}}$, $\beta_{s}=\sqrt{\gamma_{s}^{p}}$; $\Delta_{ij}$ represents the ion-covalent interaction that is considered negligible here for Van der Waals interactions play the leading role in this work.

# Supplementary Note 4. Molecular dynamic simulations

We conducted all-atom molecular dynamics (MD) simulations of two interface systems involving metal-organic frameworks (MOF) and polymers of intrinsic microporosity-1 (PIM-1 or TR-PIM) using the robust GROMACS 2020 program suite ^[1]^. A hybrid force field scheme was adopted, in which the UFF4MOFII force field ^[2–4]^ was applied to describe the bonded and non-bonded interactions within the MOF framework, while GAFF2 ^[5]^ was assigned to all polymer components, ensuring compatibility across organic and inorganic phases. The polymer molecules were parameterized with the GAFF2 force field, alongside AM1-BCC atomic charge, while the MOF slab models were meticulously parameterized using the UFF4MOFII force field and REPEAT atomic charge ^[6]^. All topology files for these molecules and MOF materials were seamlessly generated through the AuToFF web server [**Ref**: <https://cloud.hzwtech.com/web/product-service?id=36>.].

To construct a physically realistic polymer matrix prior to interfacial simulations, we employed a solution-casting-inspired relaxation protocol. A total of 45 polymer chains, each consisting of 8 to 10 repeating units, were generated based on the chemical structure of TR-PIM and PIM-1 and capped with hydrogen atoms to eliminate terminal high-energy sites. For each polymer chemistry (PIM-1 and TR-PIM), several distinct low-energy chain conformations were first generated and then randomly replicated and placed in the simulation cell so that the resulting amorphous phase contained multiple independent conformers and local packing environments, rather than a single special chain geometry. The polymer chains were randomly packed into a periodic simulation box along with explicit chloroform (CHCl_3_) molecules to reproduce the polymer solution environment. The initial box size was chosen such that the overall mass density of the polymer/solvent mixture was approximately 0.5 g/cm^3^, providing sufficient free volume for chain rearrangement during equilibration. All components were described using the GAFF2 force field, and atomic partial charges were assigned using the AM1-BCC scheme. The system was first equilibrated under NPT conditions (300 K, 1 bar) for 2 ns to allow uniform dispersion of polymer chains within the solvent phase. Subsequently, the chloroform molecules were gradually removed in 5–10 separate stages, each involving the deletion of 10–20% of the remaining solvent molecules based on their center-of-mass distance from the box boundaries. After each deletion step, the system was equilibrated under NVT conditions at 300 K for 200–500 ps, allowing the polymer chains to relax and reorganize in response to solvent loss. This sequential evaporation and relaxation process was repeated until all solvent molecules had been removed. Following complete solvent removal, the dry polymer structure was subjected to a final 2 ns NPT equilibration at 1 bar and 300 K to ensure structural stability and convergence.

From the equilibrated MOF–polymer interface models, the effective polymer-phase densities are approximately 0.91 g/cm^3^ for PIM-1 and 1.14 g/cm^3^ for TR-PIM. Literature reports for PIM-1 give bulk densities of roughly 0.90–1.00 g/cm^3^ from molecular simulations and about 1.0–1.3 g/cm^3^ at 25 °C for solution-cast membranes, depending on casting and conditioning protocols. In our own measurements on solution-cast films, the bulk densities of PIM-1, PIM-300TR, and PIM-350TR are 0.974, 1.073, and 1.108 g/cm^3^, respectively. The simulated polymer densities are therefore consistent with both the literature range and our experimental values, and they reproduce the experimentally observed densification upon thermal cyclotrimerization, supporting the physical realism of the glassy polymer environment generated by the solvent-evaporation protocol.

MAF-stu-1 slabs were generated by cleaving the bulk crystal along the (001) lattice plane. The slab was placed at the center of the simulation cell along the z-direction, with two vacuum layers (6.6 nm each) appended on both sides to eliminate spurious interactions between periodic images and to accommodate interfacial polymer relaxation. For the MAF-stu-1@PIM-1 system, the final box dimensions after CHCl_3_ removal and NPT equilibration are 9.3097 × 8.0624 × 8.0921 nm^3^. For the MAF-stu-1@TR-PIM system, the final box dimensions are 9.3097 × 8.0624 × 6.9673 nm^3^. In both cases, the shortest box length (~7 nm) is more than five times larger than the non-bonded cut-off (1.2 nm), and thus comfortably exceeds the commonly used criterion that each box dimension should be at least three times the cut-off to minimize artefacts from periodic images. Each box included two vacuum layers, and was carefully packed with PIM-1 and TR-PIM molecules, replicating the mole ratios used in our experimental setup. We first subjected these structures to energy minimization and proceeded with a comprehensive 20 ns MD simulation in the constant NVT ensemble, capturing trajectory data every 10 ps. For structural and energetic analysis (e.g., RDFs and MOF–polymer interaction energies), the 20 ns trajectory (2002 frames) was divided into several non-overlapping time blocks, and block-averaged means and standard deviations were computed to quantify the variability along the trajectory. Throughout the simulation, a precise temperature of 298.15 K was maintained using the velocity-rescale thermostat ^[7]^, with a coupling time constant of 1 ps, providing stable thermal control while preserving canonical ensemble sampling. Periodic boundary conditions were imposed in all spatial directions to mimic bulk-like behavior and avoid edge effects.

Electrostatic interactions were calculated using the particle-mesh Ewald (PME) method, and both van der Waals and short-range electrostatics were truncated at 1.2 nm. Interfacial structural features were characterized through the calculation of one-dimensional number density distributions, radial distribution functions (RDFs), and hydrogen bond statistics using GROMACS analysis tools, enabling spatially resolved evaluation of polymer–MOF interactions. Polymer infiltration into the MOF surface and local coordination features were visualized using molecular rendering software. Finally, the structures were vividly rendered with the Visual Molecular Dynamics (VMD) program, showcasing the intricate details of these interactions. This comprehensive approach not only enhances our understanding of MOF/PIM interfaces but also paves the way for future innovations in material science.

To quantify the non-bonded interaction components across the MOF–polymer interface, the interaction energies were decomposed into van der Waals (Lennard-Jones) and electrostatic (Coulombic) contributions based on post-processing of the simulation trajectories. Energy terms between the MOF and polymer domains were computed by defining distinct interaction groups, enabling the extraction of intermolecular interaction energies throughout the simulation. The resulting ensemble-averaged values provide quantitative insight into the dominant non-covalent forces governing interfacial behavior. The non-bonded interactions included vdW and electrostatic interactions, which are described by Eq. S8 and S9, respectively.

$E_{\text{LJ}}\left( r_{ij} \right)=4 \varepsilon_{ij}\left[ \left( \frac{\sigma_{ij}}{r_{ij}} \right)^{12}-\left( \frac{\sigma_{ij}}{r_{ij}} \right)^{6} \right]$ (S8)

Here, *r_ij_* represents the distance between atoms *i* and *j*, *ɛ_ij_* denotes the energy parameter between atoms *i* and *j*, and *σ_ij_* denotes the atomic size parameter between atoms *i* and *j*.

$E_{c}\left( r_{ij} \right)=\frac{q_{i}q_{j}}{4\pi\varepsilon_{0} \varepsilon_{r} r_{ij}}$ (S9)

Here, *r_ij_* represents the distance between atoms *i* and *j*; *q_i_* and *q_j_* represent the atomic charges of atoms *i* and *j*, respectively; *ɛ_0_* is the vacuum dielectric constant; and *ɛ_r_* is the relative dielectric constant.

For different types of atoms, the Lorentz-Berthelot mix rules were adopted for the vdW interactions, as given by Eq. S10. A uniform cutoff distance of 1.2 nm was used for both van der Waals and short-range electrostatics.

$\sigma_{ij}=\frac{1}{2}\left( \sigma_{ii}+\sigma_{jj} \right);$ $\varepsilon_{ij}=\left( \varepsilon_{ii} \varepsilon_{jj} \right)^{1/2}$ (S10)

# Supplementary Note 5. Techno-economic analysis of membrane systems for post-combustion carbon capture

A two-stage membrane cascade was simulated in UniSim Design R471 integrated with an in-house customized membrane model (ChemBrane) ^[8–10]^ to assess the technological feasibility of post-combustion flue-gas CO_2_ capture under the operating basis and membrane properties specified in the main text. In this configuration, the Stage-2 feed equals the Stage-1 permeate (permeate-to-feed cascade), and the Stage-2 permeate is taken as the CO_2_-enriched product stream, while the overall recovery target is enforced by adjusting the Stage-1 and Stage-2 membrane areas (*A*_1_, *A*_2_); the Stage-2 retentate is recycled to the Stage-1 feed. The feed stream was specified as 446.1 kmol h⁻¹ at 298 K and 100 kPa, with a CO_2_/N_2_ molar ratio of 1:9 (dry basis) and compressed to 200 kPa upstream of Stage-1 and the Stage-1 permeate is recompressed to 200 kPa upstream of Stage-2. Both permeate sides were maintained at 10 kPa. A vacuum-assisted sweep is applied on the permeate sides to sustain the transmembrane driving force while avoiding excessively deep vacuum, and coolers restored the gas temperature to 298 K, ensuring isothermal operation. Because the flue gas is compressed upstream of Stage-1, feed compression is explicitly modeled. Within this note, permeate pressure is fixed at 10 kPa. Transport follows the solution-diffusion description implemented in ChemBrane with constant permeance; pressures and compositions are taken as bulk values on each side of the membrane. In all simulations, the specified separation targets (overall CO_2_ recovery and product purity) are enforced by adjusting the membrane areas (*A*_1_, *A*_2_); ADJ-1 controlled the overall recovery and ADJ-2 the product purity. Recycle convergence was implemented via RCY-1 with tolerance of 1×10⁻⁶. Permeate pressures are treated as fixed setpoints within each case and varied parametrically across scenarios to evaluate the trade-off between membrane area and power demand. As expected, lower permeate pressures decrease the membrane area required for a given separation at the expense of higher vacuum-pump energy. Transport follows the solution-diffusion description implemented in ChemBrane with constant permeance; pressures and compositions are taken as bulk values on each side of the membrane.

For cost estimation, we followed prior TEA frameworks to size and cost the compressors, vacuum pumps, and membrane modules, which dominate capital expenditures ^[11,12]^. Equipment purchased costs for rotating machinery (compressors/pumps) were estimated using CAPCOST 2012 correlations (S11) ^[13]^:

$\log_{10} C_{p}^{0}=K_{1}+K_{2}\log_{10} \left( Q \right)+K_{3}\left[ \log_{10} Q \right]^{2}$ (S11)

where *Q* is the shaft power (kW) and (*K*_1_, *K*_2_, *K*_3_) = (2.289, 1.360, 0.1027) for standard carbon-steel centrifugal units.

The annual electricity operating cost (OPEX) is computed as (S12):

$\text{OPEX}=P_{\text{total}}\cdot t_{\text{annual}}\cdot C_{\text{elec}}$ (S12)

where *P*_total_ is the total shaft/electric power (kW) of compressors and vacuum pumps, *t*_annual_ is operating hours per year (8000 h/year), and *C*_elec_ is the electricity price (e.g., $0.10/kWh) ^[14,15]^.

The corresponding bare-module cost (*C_BM_*) is (S13):

$C_{BM}=C_{p}^{0}\cdot F_{BM}$ (S13)

where *F_BM_* is the bare-module factor for the selected compressor/pump type (e.g., centrifugal CS equipment) and *C_P_^0^* is the purchased cost of a single compressor/vacuum pump from CAPCOST correlation (USD).

The membrane capital cost is (S14):

$C_{\text{mem}}=A_{\text{total}}\cdot C_{\text{mem}}^{\text{unit}}$ ; $A_{\text{total}}=A_{1}+A_{2}$ (S14)

where A_1_ and A_2_ are the Stage-1 and Stage-2 membrane areas (m^2^), and $C_{\text{mem}}^{\text{unit}}$ is the unit membrane price (50-55 USD/m^2^, see Table 5). A 5-year membrane lifetime is assumed ^[15]^, with replacement handled via capital recovery.

The total module capital (*C_TM_*) is then estimated as (S15)：

$C_{TM}=1.18\cdot\left( C_{BM}+C_{\text{mem}} \right)$ (S15)

where the factor 1.18 consolidates installation, piping, and instrumentation. Consistent with prior studies ^[16]^, capital accounting is restricted to compressors, vacuum pumps, and membrane modules; auxiliaries (e.g., coolers, mixers) are excluded.

Annual capital-related cost (CRC) is (S16)：

$\text{CRC}=\alpha\cdot C_{TM}$ (S16)

with α the capital recovery factor calculated from the project lifetime and interest rate (e.g., 20 years and 7%).

Using the product flow rate in kmol/h, the annual captured CO_2_ (ton/year) is (S17):

$\text{Annual }\text{CO}_{2}=F_{\text{product}}\cdot t_{\text{annual}}\cdot M_{\text{CO}_{2}}\cdot\frac{1}{1000}$ (S17)

and the resulting specific capture cost is (S18)：

$\text{Capture Cost (USD}\text{/}\text{ton)}=\frac{\text{OPEX}+\text{CRC}}{\text{Annual }\text{CO}_{2}}$ (S18)

Together, these equations define the annualized capital cost (CRC), operating cost (OPEX), and the resulting specific CO_2_ capture cost (USD/ton), which are reported for each scenario. Modeling assumptions include steady-state operation, isothermal conditions, constant permeance, bulk-average pressures and compositions, and the neglect of concentration polarization and pressure drop effects.


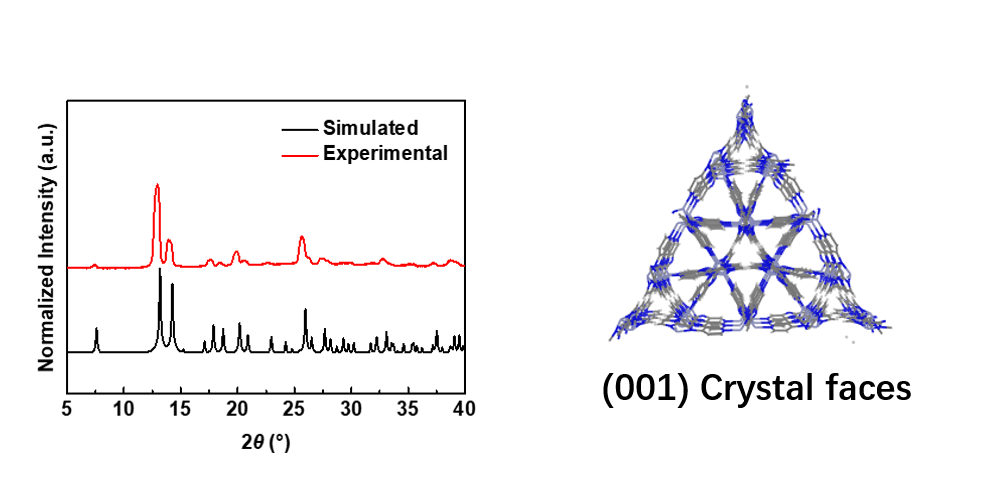


# Supplementary Figure 1. PXRD patterns and crystal structure of MAF-stu-1


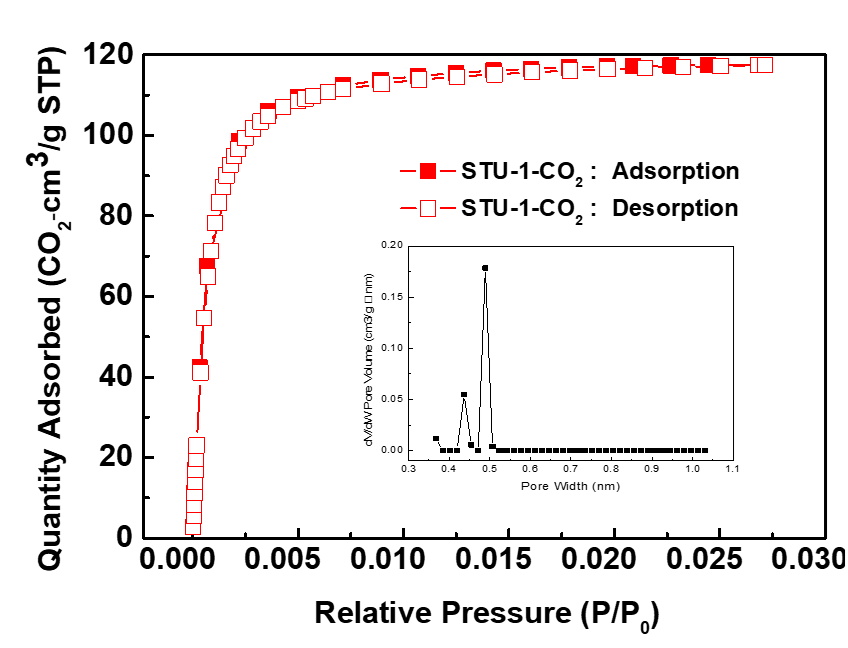


# Supplementary Figure 2. CO_2_ adsorption isotherm under 273 K and the pore size distribution, based on the DFT model.

# Supplementary Figure 3. N_2_ adsorption isotherm under 298 K

**
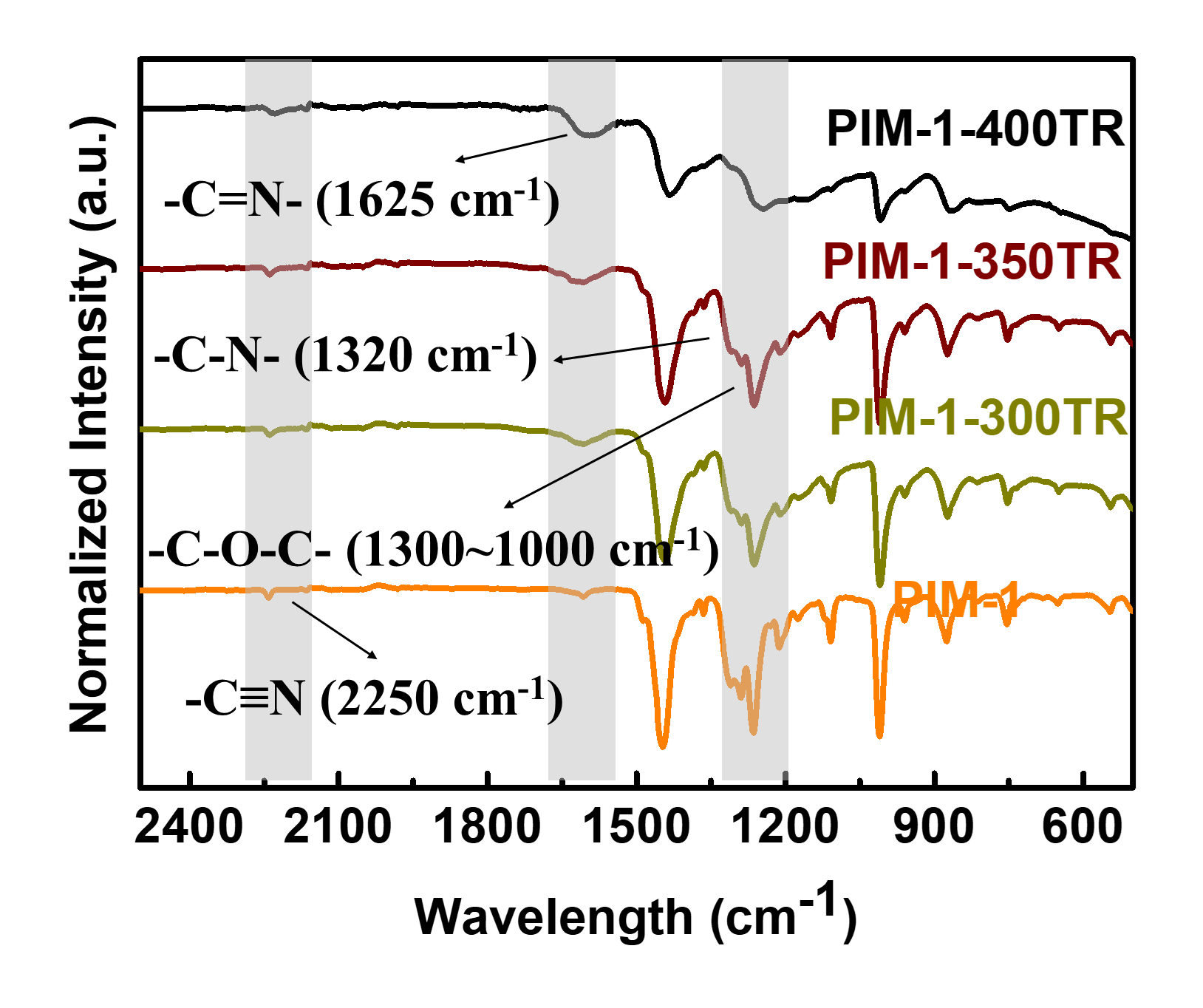
**

# Supplementary Figure 4. FTIR measurement on PIM-1 thermally treated under 300, 350 and 400 °C.


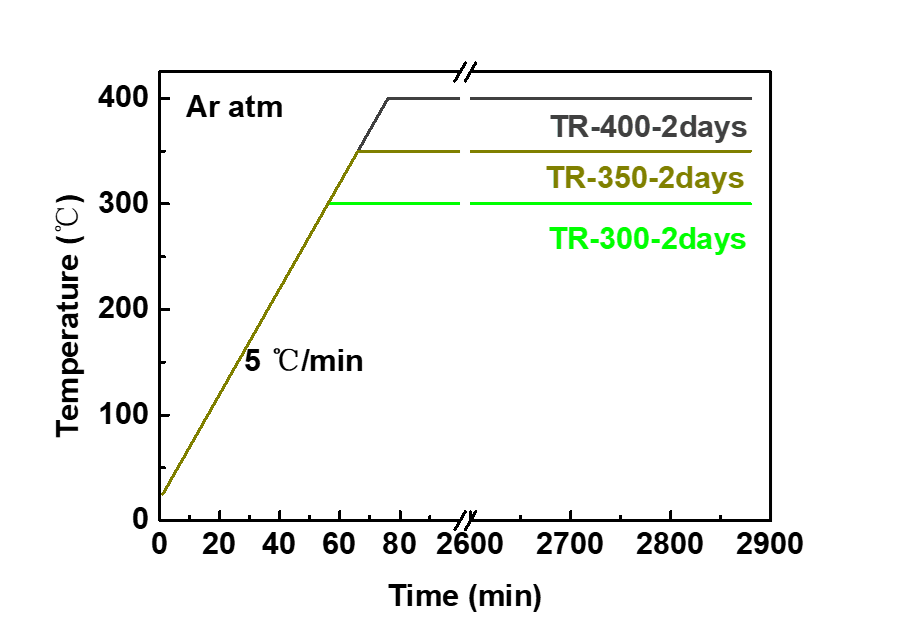


# Supplementary Figure 5. Thermal rearrangement protocol under 300, 350 and 400 °C.


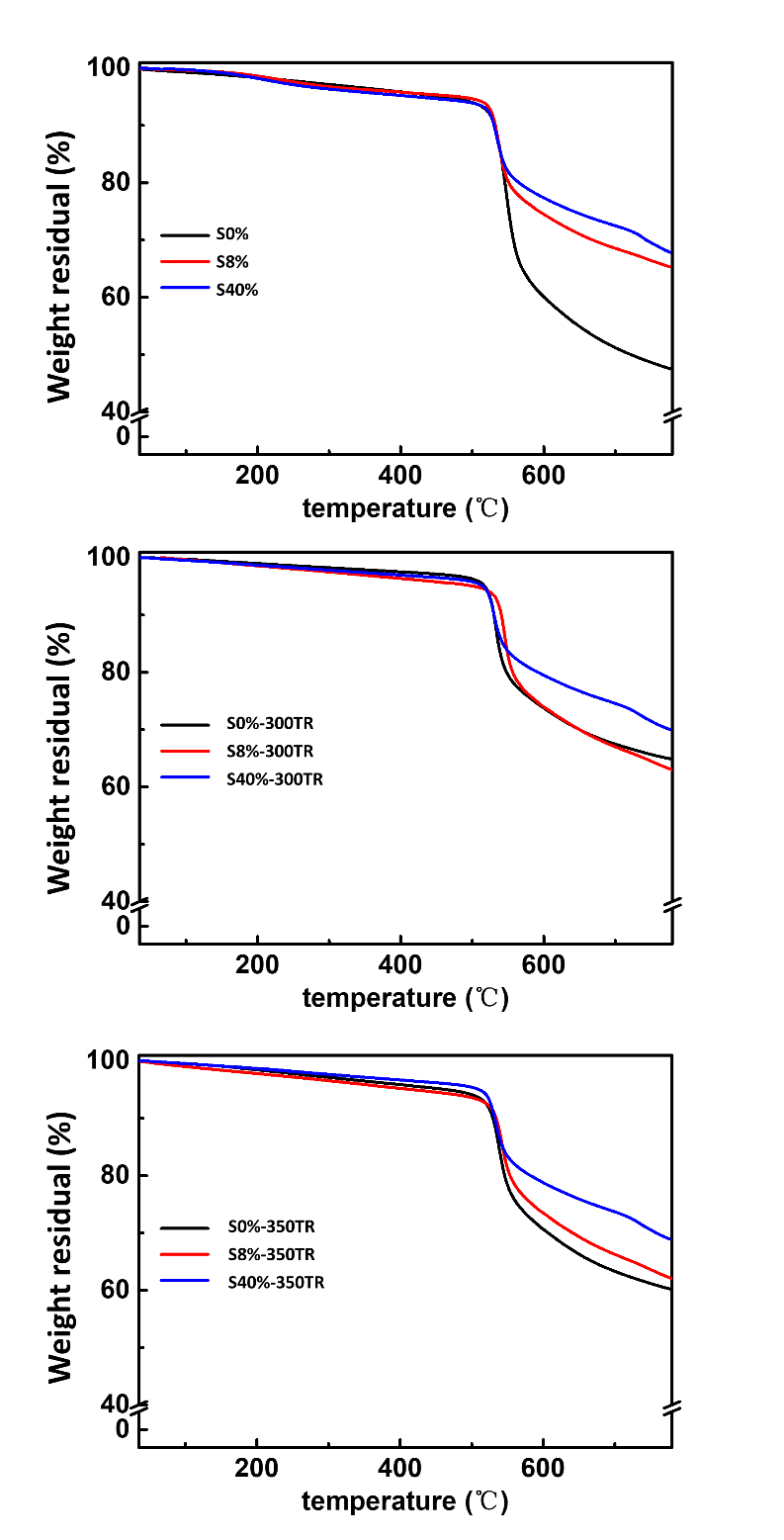


# Supplementary Figure 6. TGA analysis on pristine PIM-1 and MMMs pre and post-thermal rearrangement at different temperatures.


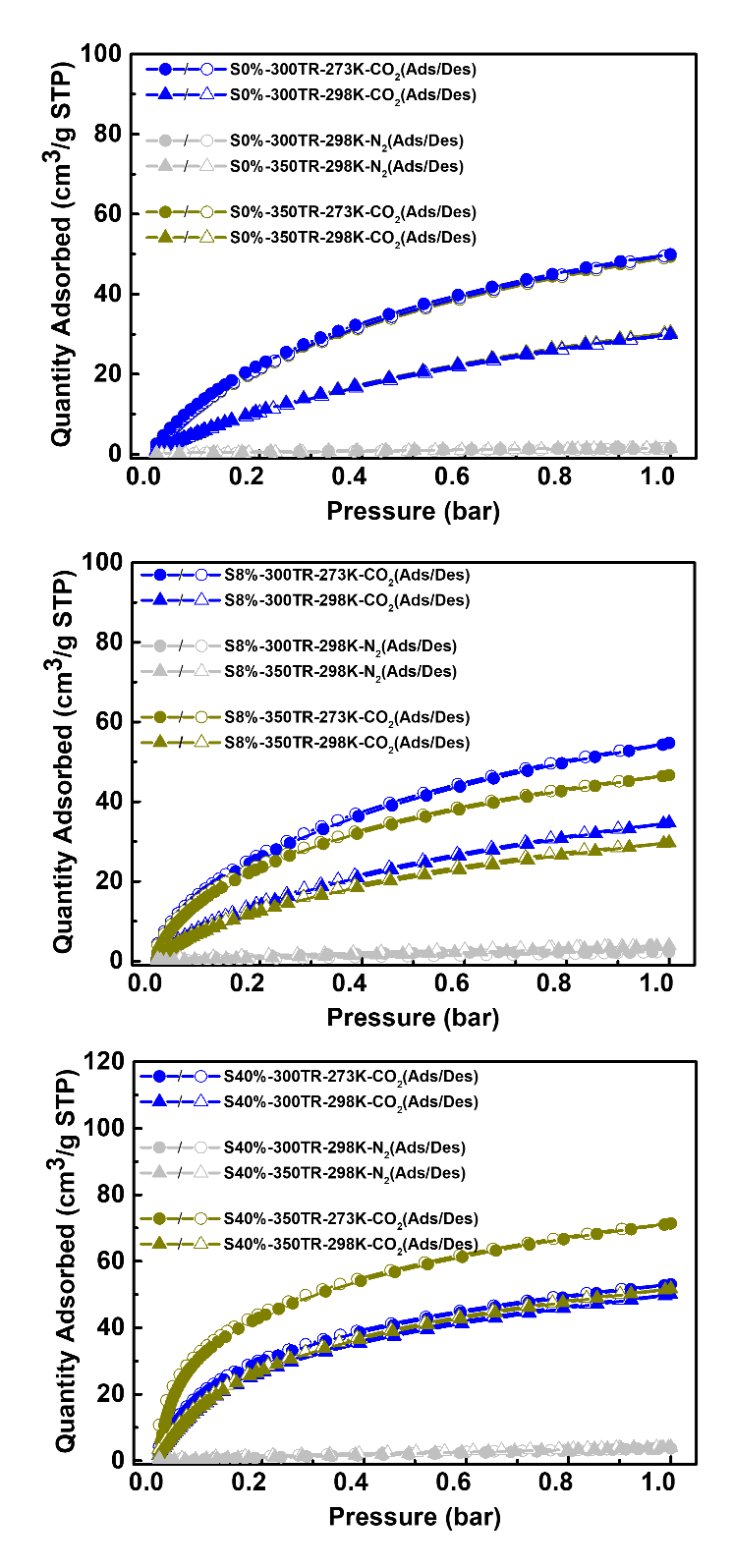


# Supplementary Figure 7. CO_2_ and N_2_ adsorption isotherm under different temperatures.


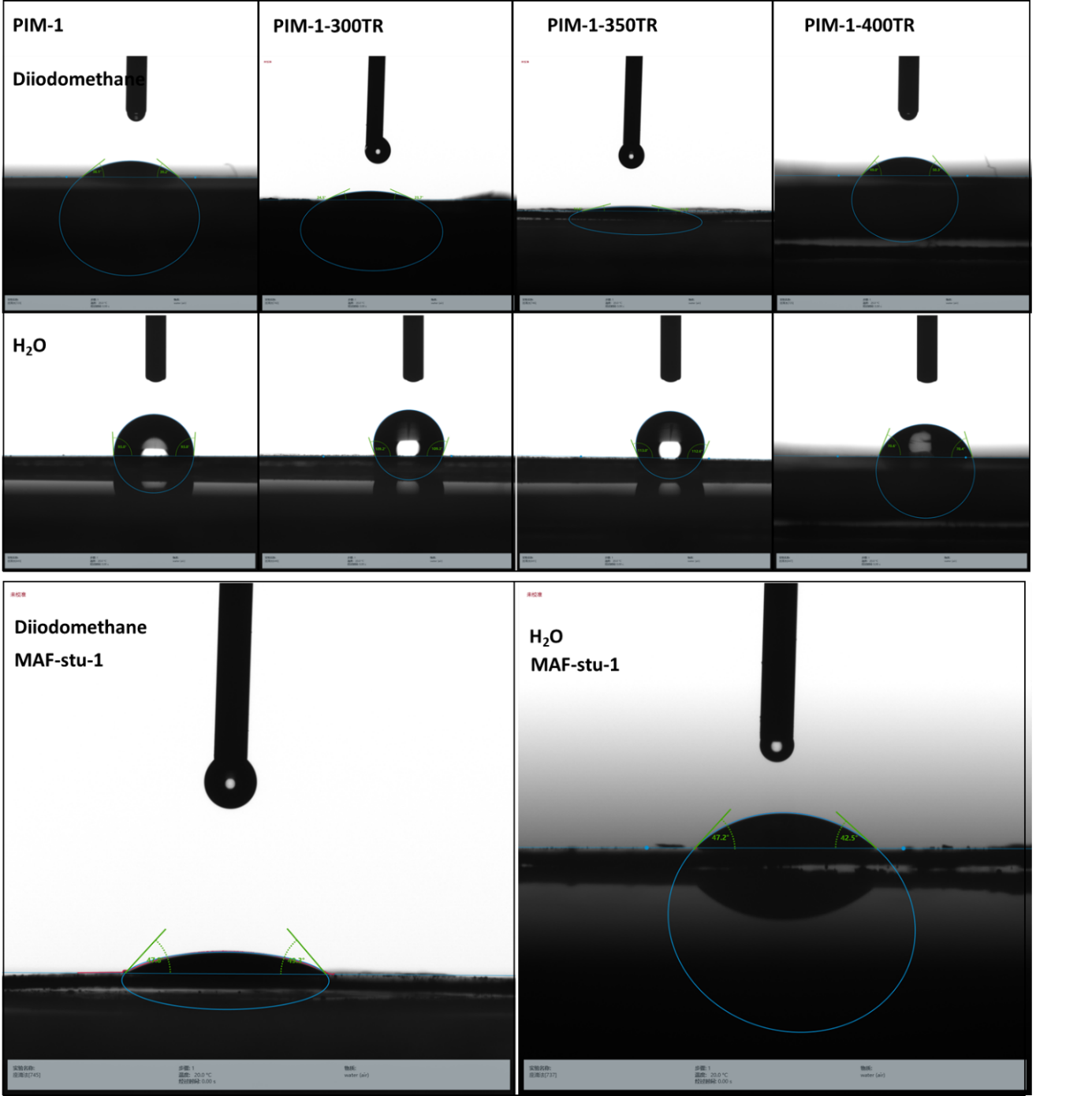


# Supplementary Figure 8. Contact angle measurements on MAF-stu-1 and PIM-1 polymer thermally treated under different temperatures by using water and diiodomethane.


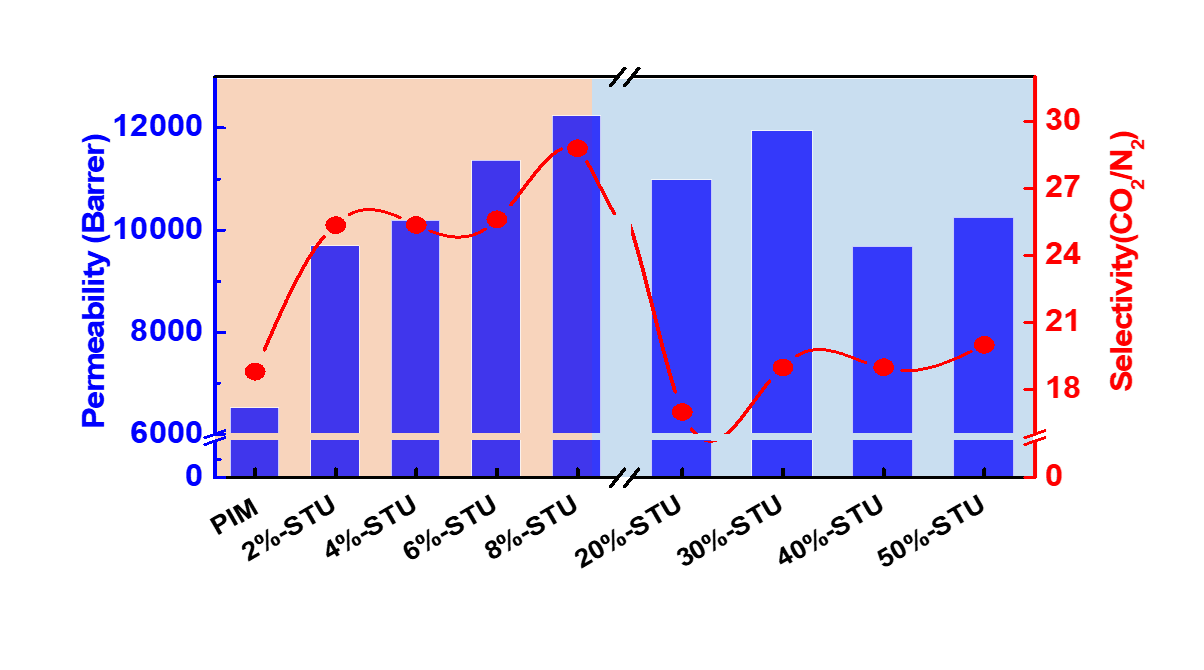


# Supplementary Figure 9. Mixed gas separation performance of MAF-stu-1@PIM-1 MMMs at different loading amounts.

**
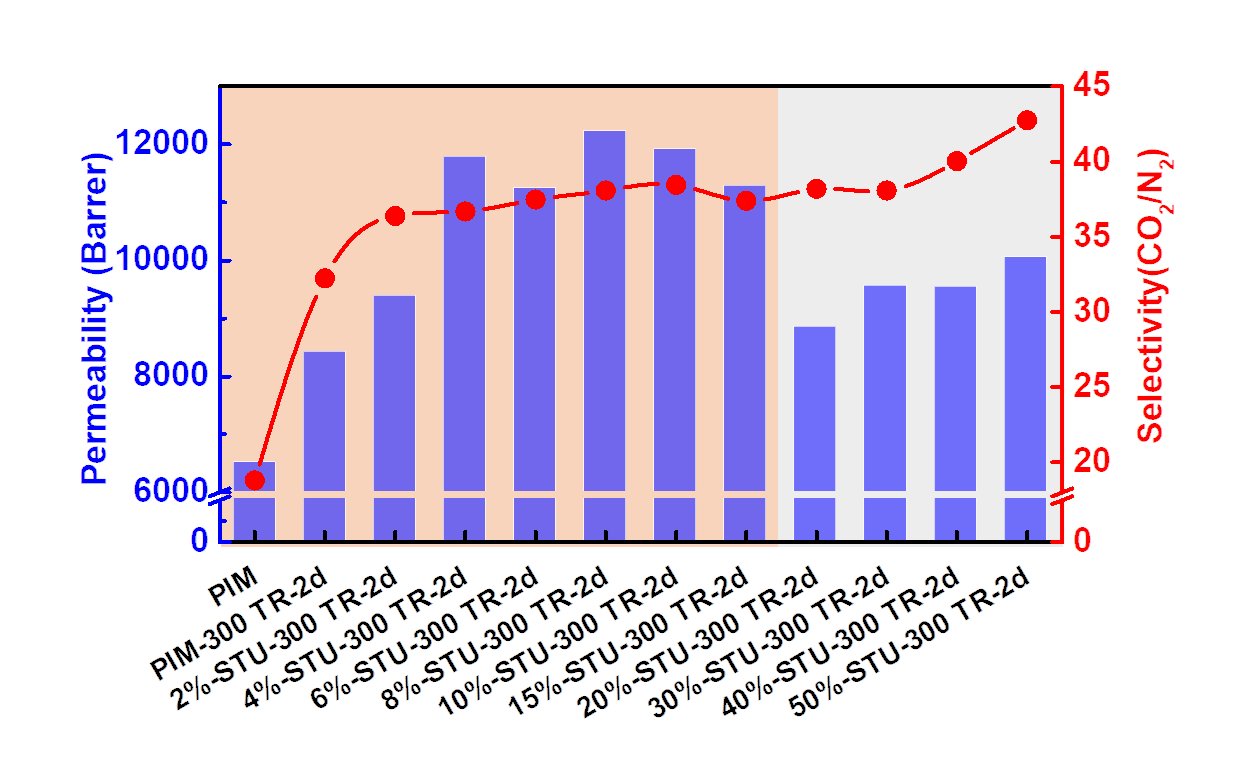
**

# Supplementary Figure 10. Mixed gas separation performance of MAF-stu-1@PIM-1 MMMs thermally treated under 300 °C for 48 hours at different loading amounts.


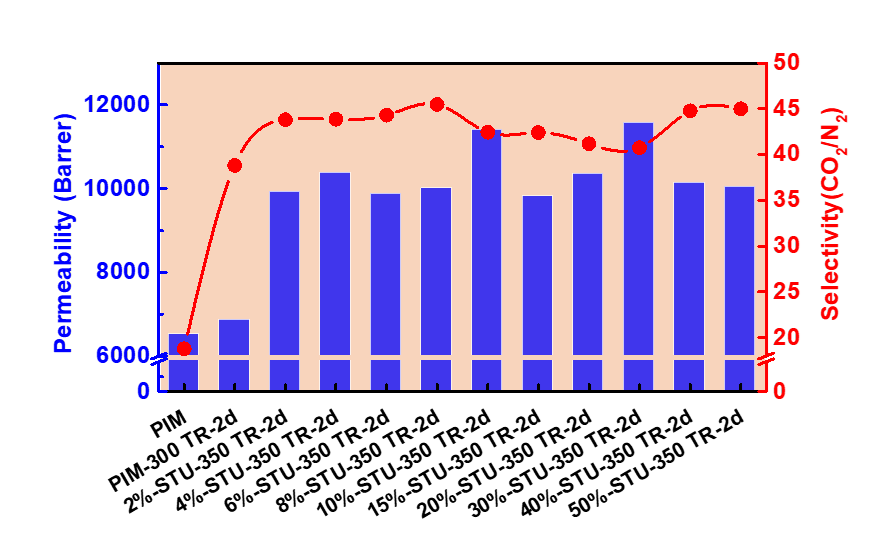


# Supplementary Figure 11. Mixed gas separation performance of MAF-stu-1@PIM-1 MMMs thermally treated under 350 °C for 48 hours at different loading amounts.

**
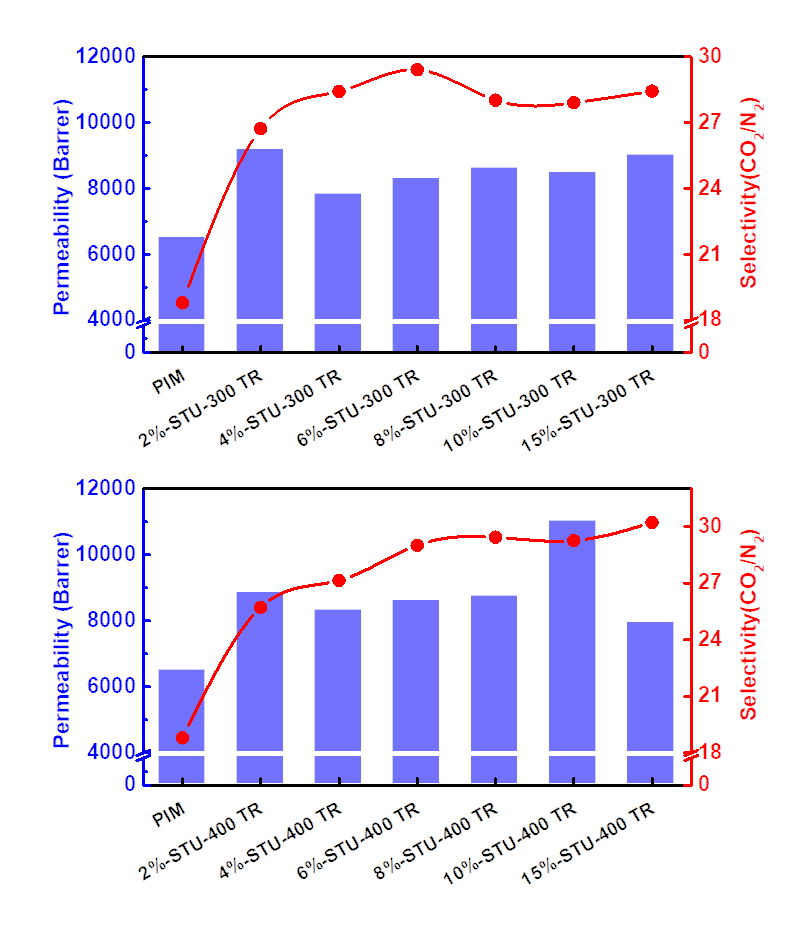
**

# Supplementary Figure 12. Mixed gas separation performance of MAF-stu-1@PIM-1 MMMs thermally treated under 300 and 400 °C for 1 hour at different loading amounts.


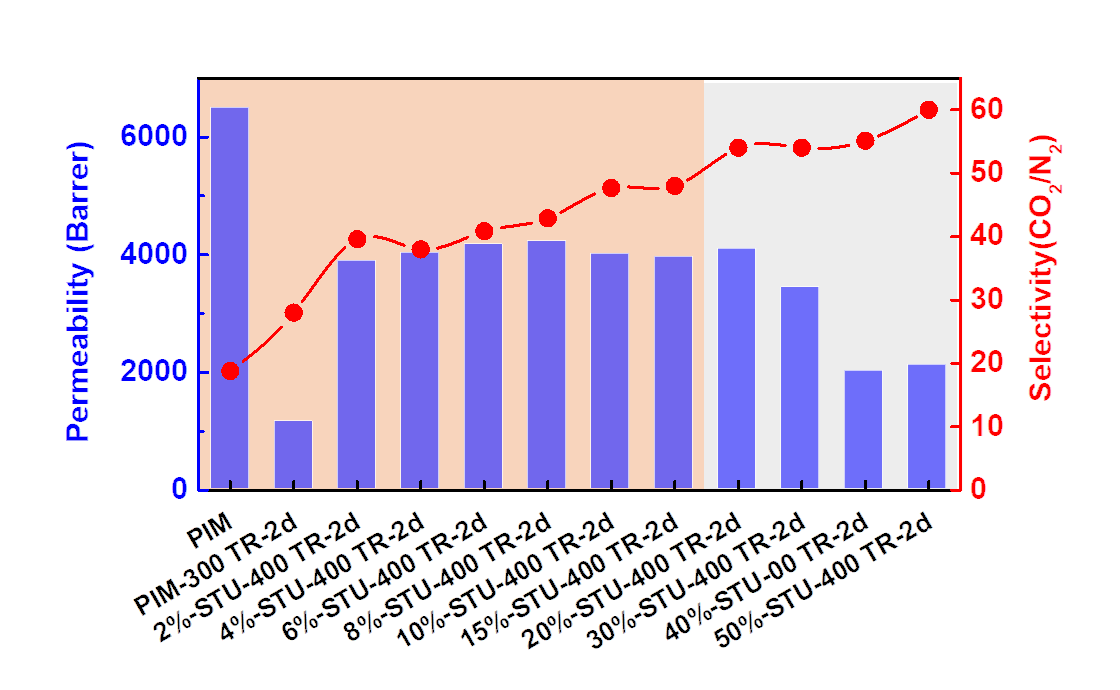


# Supplementary Figure 13. Mixed gas separation performance of MAF-stu-1@PIM-1 MMMs thermally treated under 400 °C for 48 hours at different loading amounts.

**
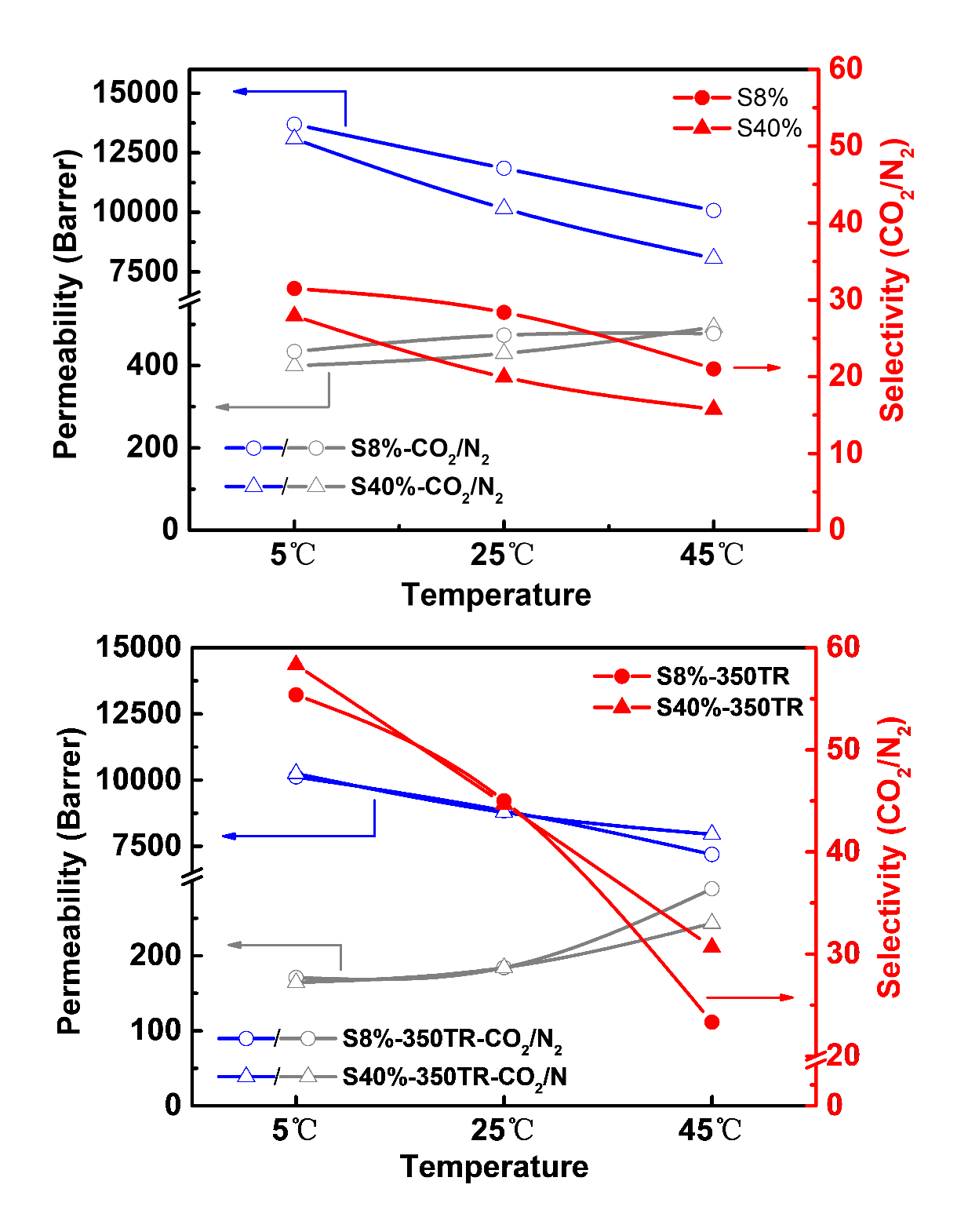
**

# Supplementary Figure 14. Temperature-dependent mixed-gas CO_2_/N_2_ permeation results on Sx and Sx-350TR membranes.


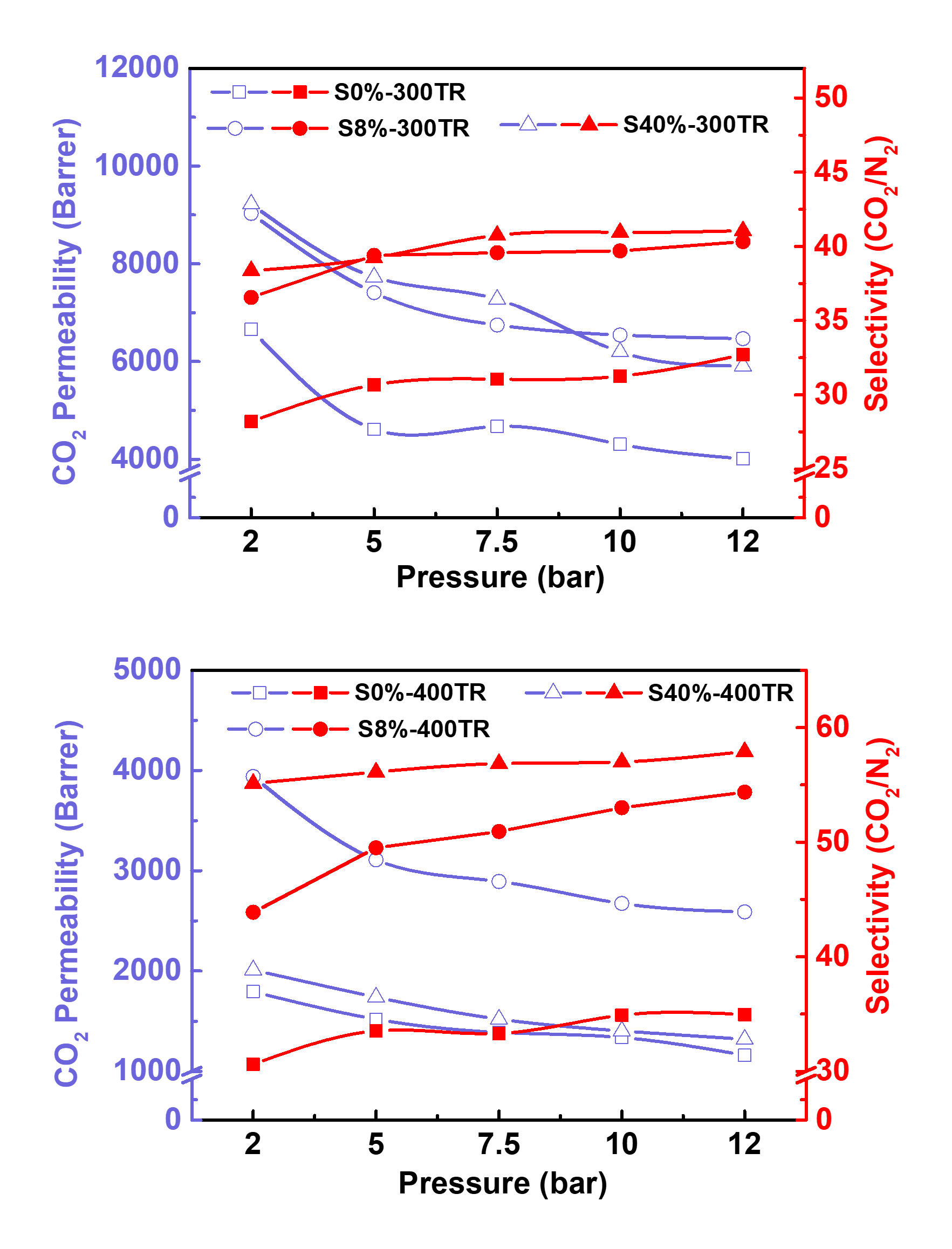


# Supplementary Figure 15. Pressure-dependent mixed-gas CO_2_/N_2_ permeation results on Sx-300TR and Sx-400TR membranes.


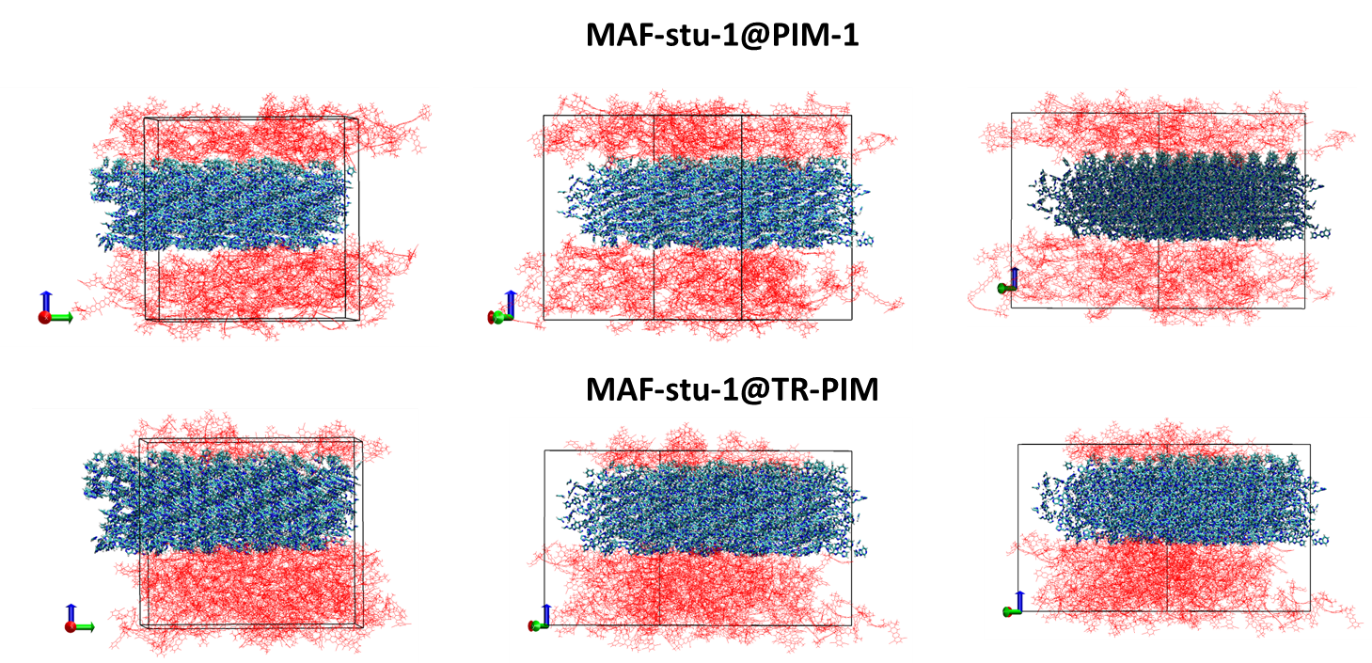


# Supplementary Figure 16. Snapshots of the MAF-stu-1/PIM-1 and MAF-stu-1/TR-PIM interface configurations.


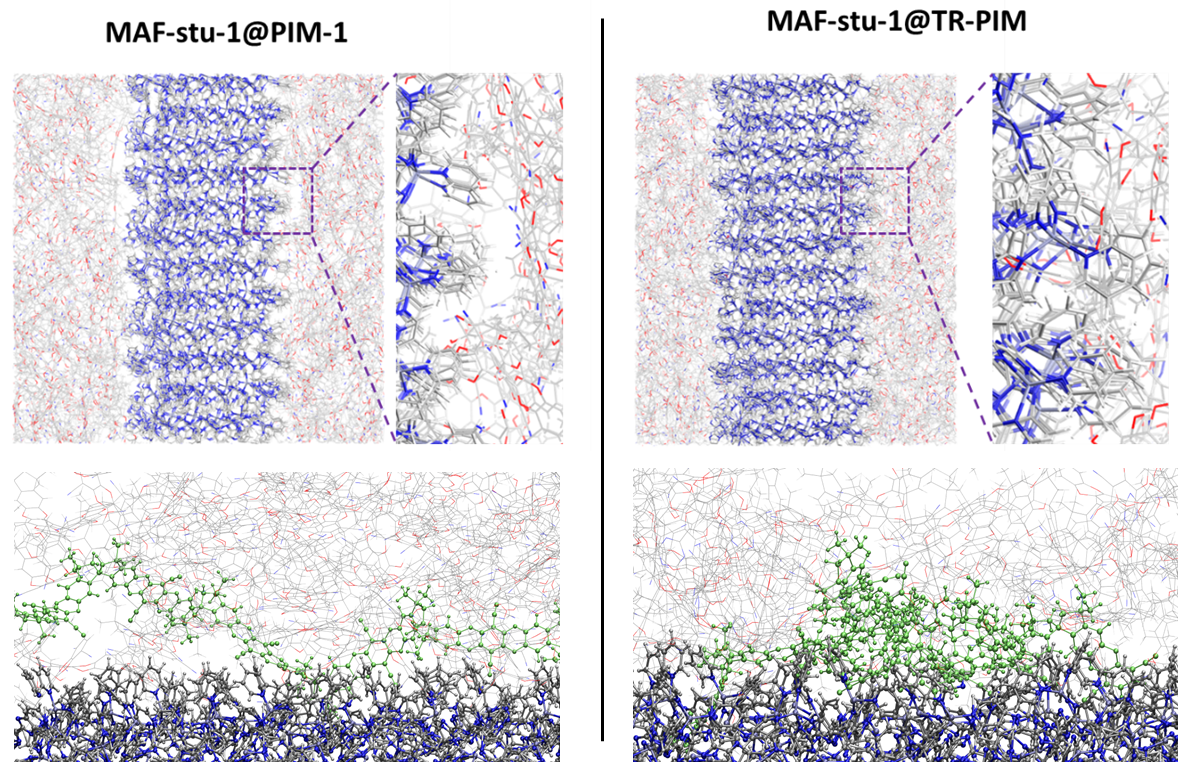


# Supplementary Figure 17. Snapshots of the MAF-stu-1/PIM-1 and MAF-stu-1/TR-PIM interface voids.


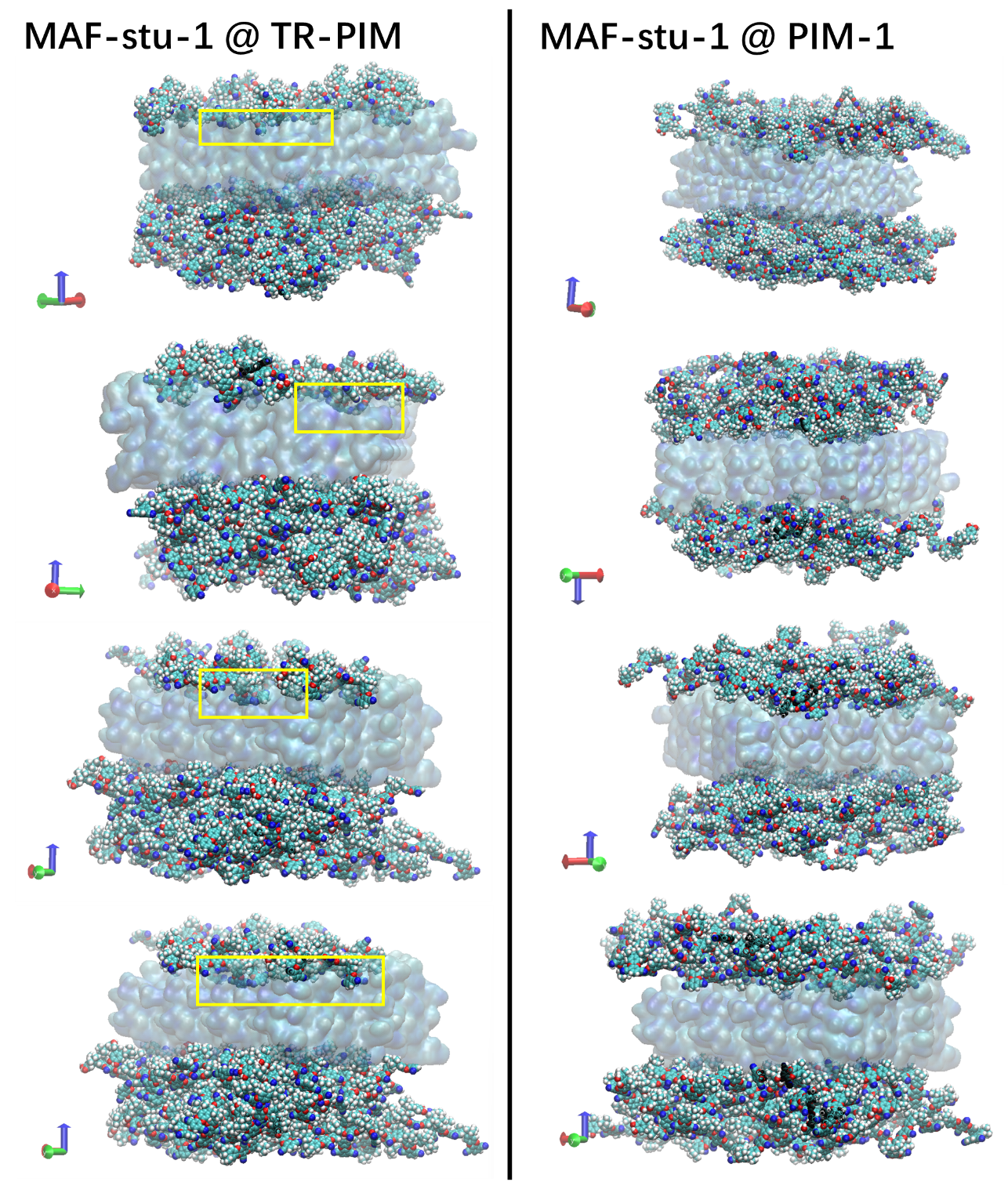


# Supplementary Figure 18. Snapshots of structural configurations of MAF-stu-1@TR-PIM and MAF-stu-1@PIM-1 obtained from molecular dynamics simulations.

(MOF frameworks are displayed in transparent surface representation, while polymer chains are shown in solid rendering. The yellow box highlights a region in the MAF-stu-1@TR-PIM system where polymer chains are positioned within the outer surface region of the MOF particle).


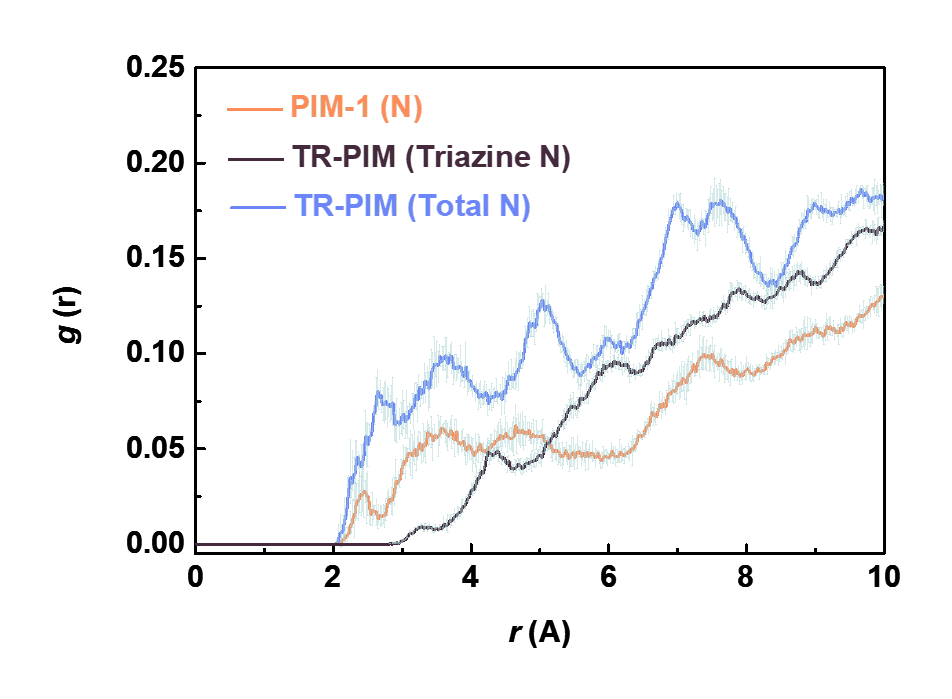


# Supplementary Figure 19. Radial distribution functions g(r) between all Zn^2+^ sites in MAF-stu-1 and N atoms from PIM-1 and TR-PIM.

(In the TR-PIM system, nitrogen atoms include both those on the original PIM framework and those incorporated into triazine rings. The RDFs are calculated over the entire trajectory and represent ensemble-averaged spatial correlations between Zn and each type of N atom across the full system, rather than specific atom pairs, and the error bars reflect the standard deviations obtained by block-averaging the 20 ns trajectory into multiple time windows.)


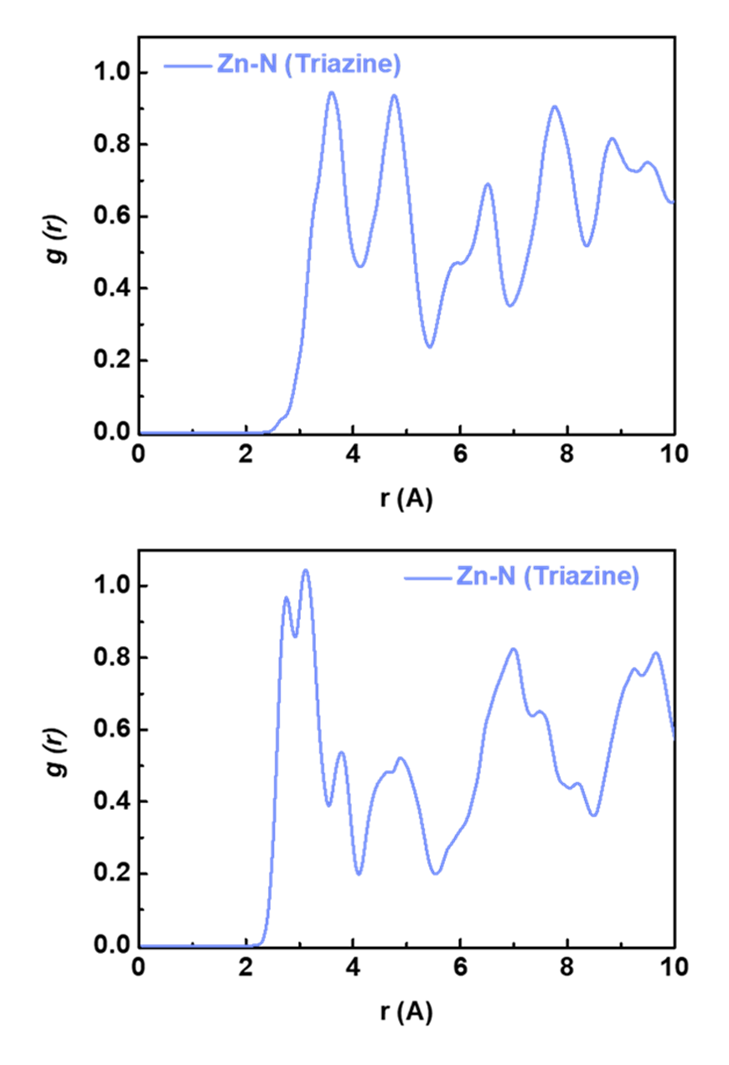


# Supplementary Figure 20. Radial distribution functions g(r) between individual Zn^2+^ sites in MAF-stu-1 and neighboring triazine nitrogen atoms from two distinct polymer segments in the TR-PIM matrix.

(Each curve represents the trajectory-averaged RDF of a single Zn–N(triazine) pair, providing local structural insight into Zn–N interactions in different interfacial regions, independent of system-wide averaging.)


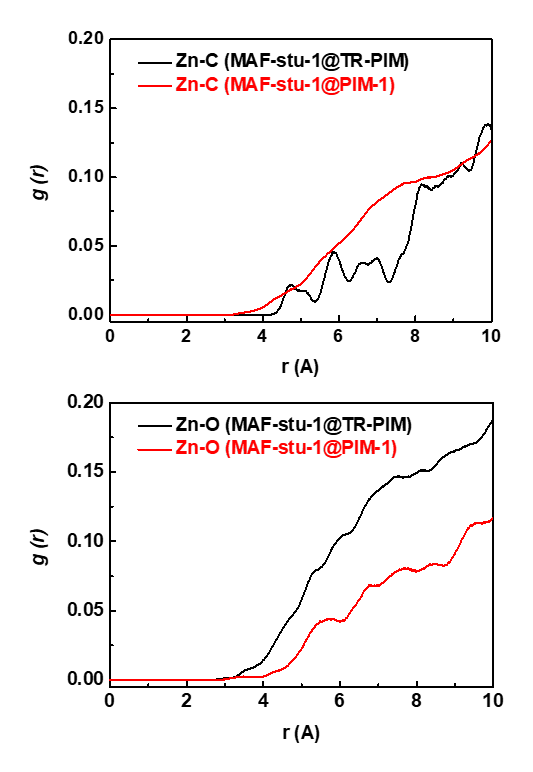


# Supplementary Figure 21. RDF between Zn²⁺ sites in MAF-stu-1 and (top) C atoms and (bottom) O atoms from the surrounding polymer segments.

(Each curve represents the ensemble-averaged Zn–C or Zn–O spatial correlation calculated over all relevant atom pairs throughout the full MD trajectory, providing insight into the non-specific interfacial interactions between Zn²⁺ and the polymer matrix.)

# Supplementary Figure 22. Time evolution of the number of hydrogen bonds formed between donor N-H groups on MAF-stu-1 and acceptor O atoms on the TR-PIM polymer backbone.

(Hydrogen bonds were identified based on a donor–acceptor distance threshold of 3.2 Å and a D–H···A angle range of >150°, evaluated across the full MD trajectory.)


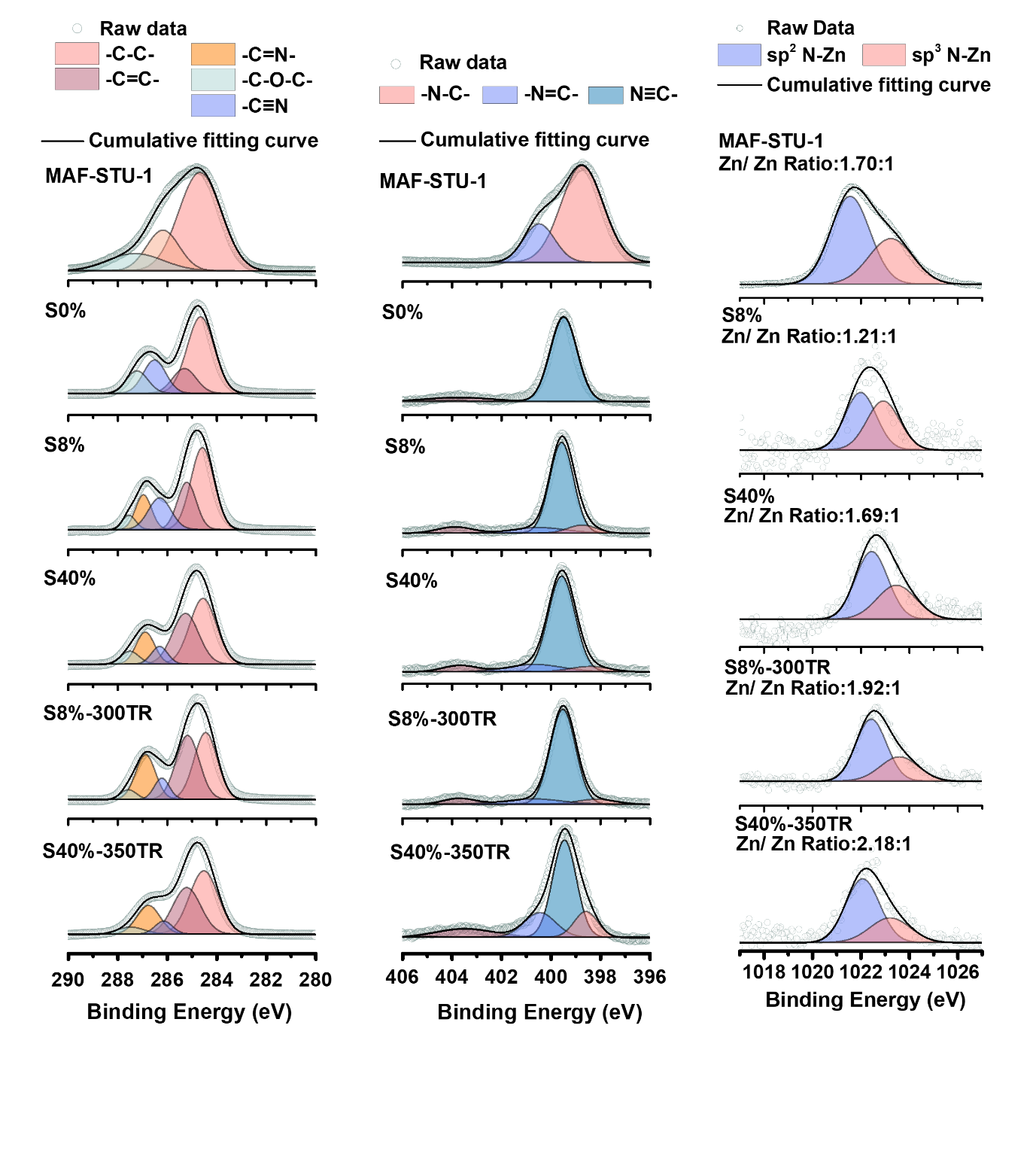


# Supplementary Figure 23. Cyclotrimerization of the -C≡N groups following thermal rearrangement, demonstrated through X-ray photoelectron spectroscopy (XPS) analysis of the Zn 2p, C 1s and N 1s spectra.


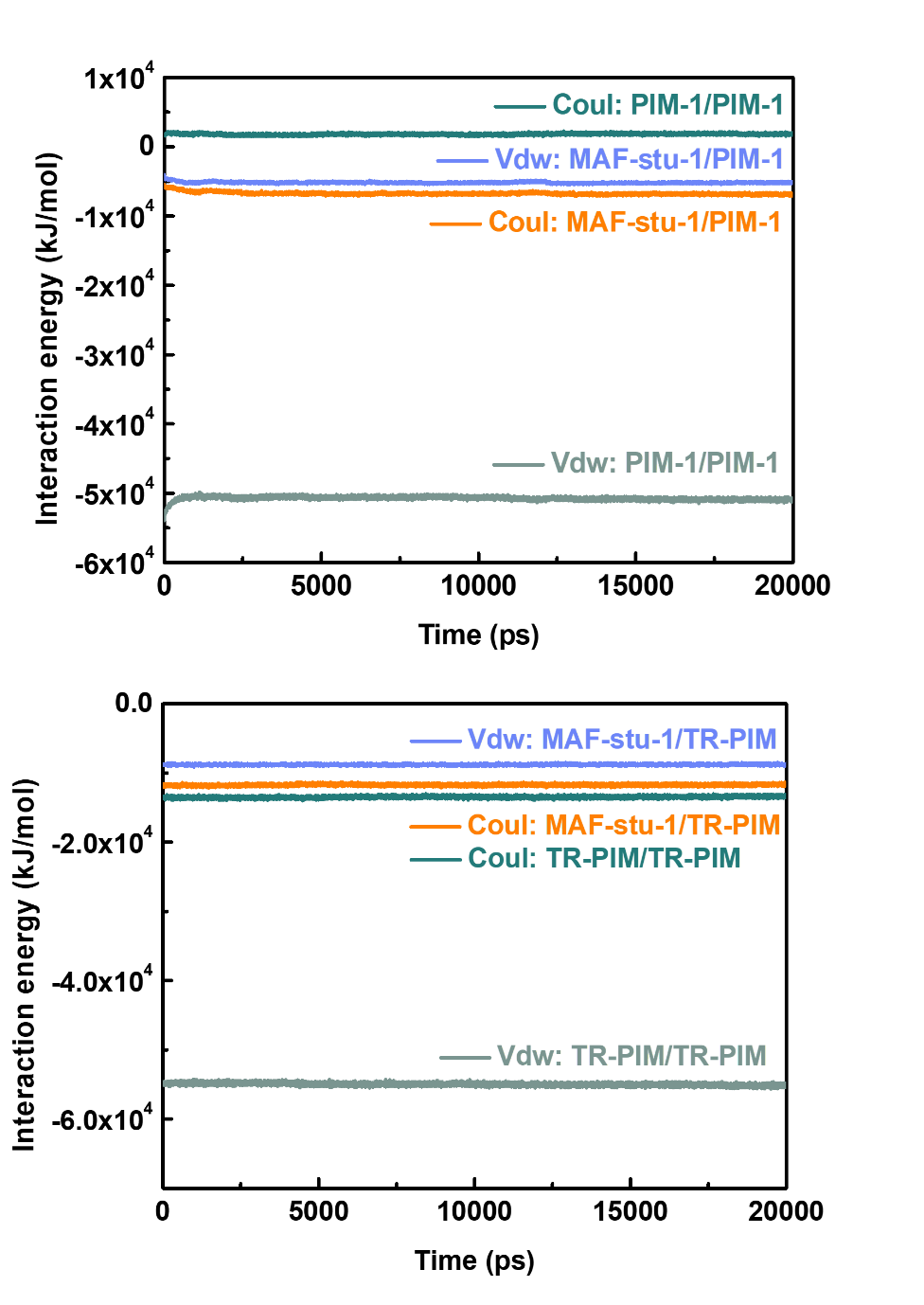


# Supplementary Figure 24. Time-dependent Coulombic and van der Waals interaction energies for the MAF-stu-1/PIM-1, MAF-stu-1/TR-PIM, PIM-1/PIM-1 and TR-PIM/TR-PIM systems.


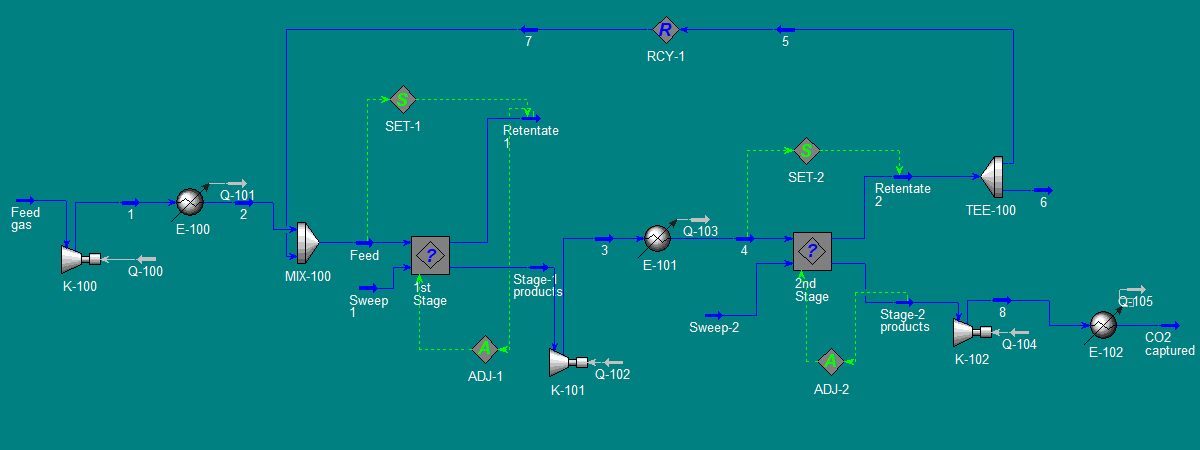


# Supplementary Figure 25. Two-stage membrane cascade with recycling, used for post-combustion CO_2_ capture process simulations.


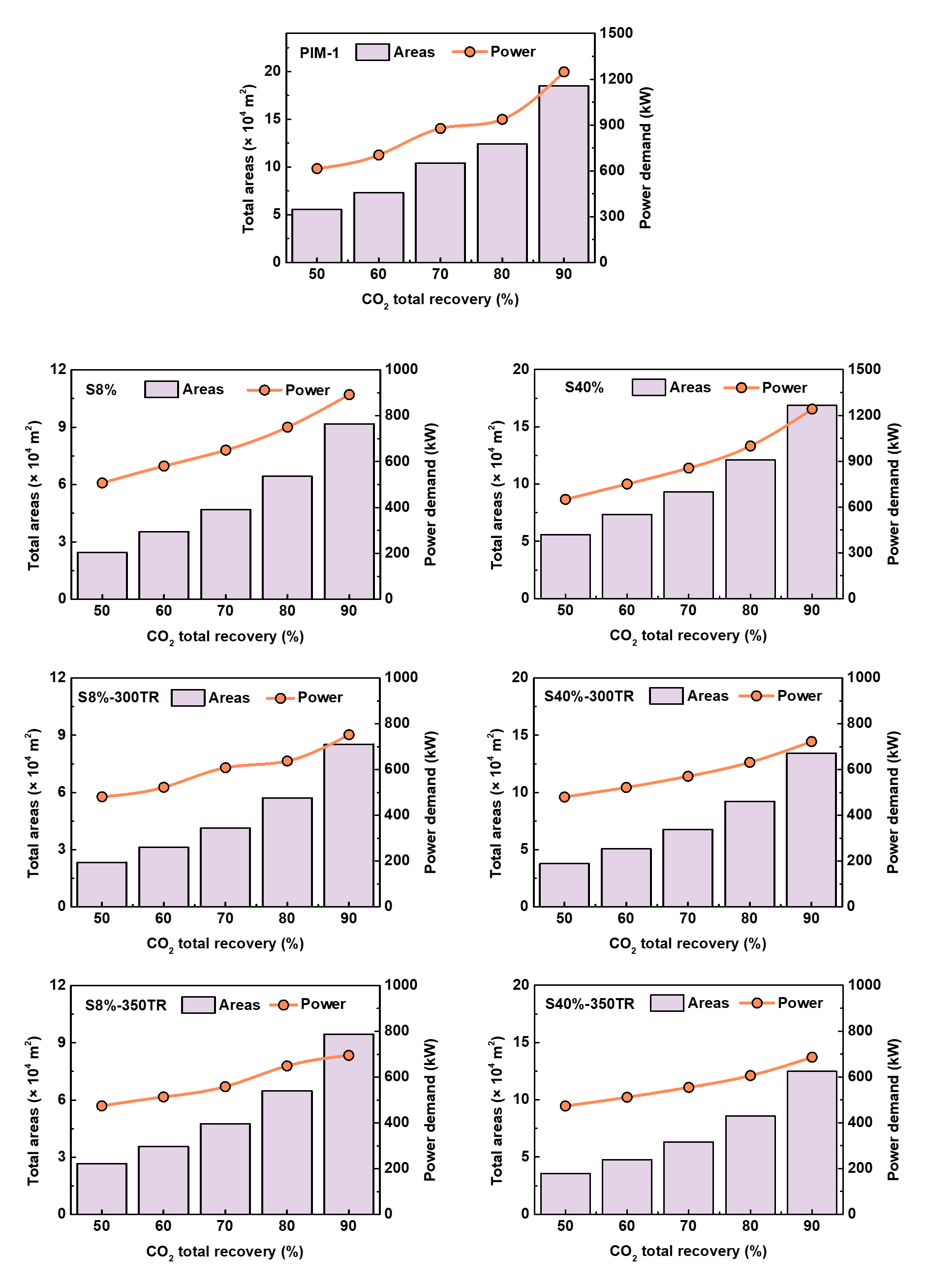


# Supplementary Figure 26. The sensitivity analysis of CO_2_ recovery on stage-wise and total membrane area, and power demand for PIM-1, MMMs, and TR-MMMs.


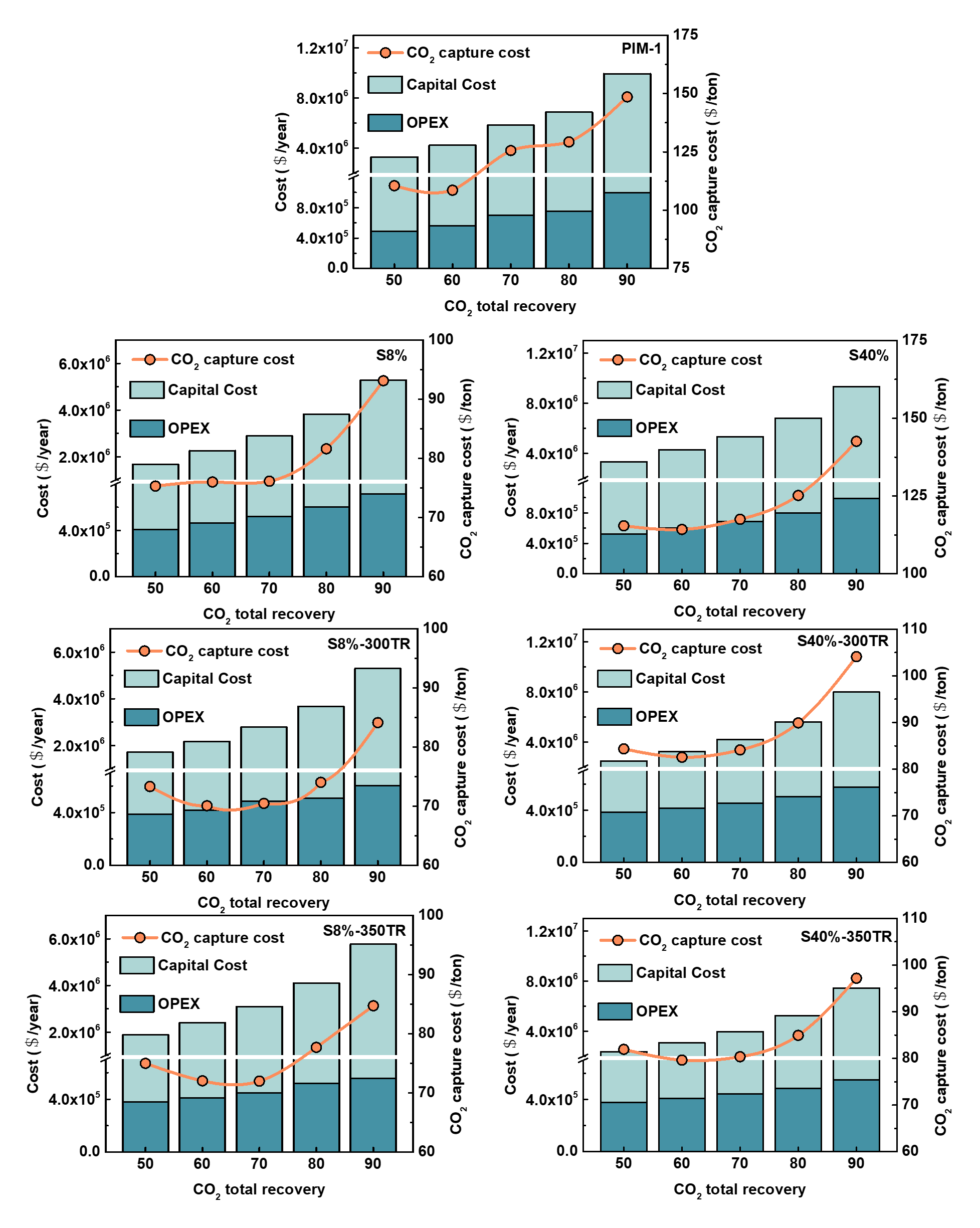


# Supplementary Figure 27. The sensitivity analysis of CO_2_ recovery on OPEX, capital cost, and annual CO_2_ captured, and specific CO_2_ capture cost for PIM-1, MMMs, and TR-MMMs.


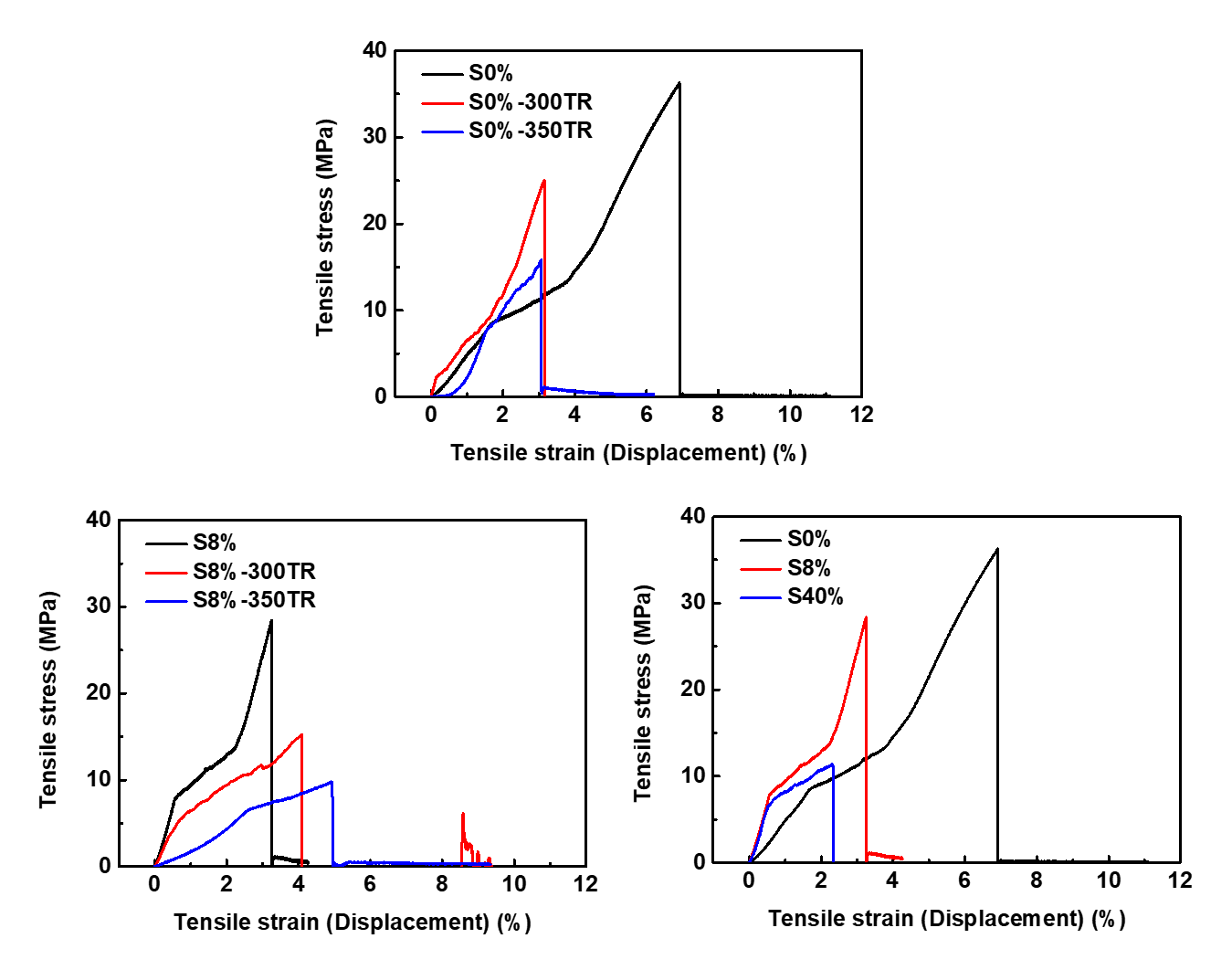


# Supplementary Figure 28. Uniaxial tensile stress–strain curves for dense, self-standing membranes of neat PIM-1 (S0%), thermally treated S0%-300TR and S0%-350TR, MMMs with 8 wt% MAF-stu-1 (S8%, S8%-300TR, S8%-350TR), and the as-cast 40 wt% MMM (S40%). All samples were tested under identical conditions at room temperature.

(For neat PIM-1, thermal cyclotrimerization stiffens the glassy network and makes it more brittle, consistent with triazine crosslinking and some high-temperature degradation. For the 8 wt% MMM, the rigid MAF-stu-1 particles increase stiffness and reduce ductility in the as-cast state, and thermal treatment further amplifies interfacial stress concentrations, so the membranes fail at lower stress, although crack deflection around the particles still allows a modest strain before fracture. For the 40 wt% MMM, the dense filler network and defect-sensitive interfaces dominate the response, causing both tensile strength and strain at break to drop relative to neat PIM-1 and S8, and after thermal treatment the samples become too brittle to be clamped and tested reproducibly.)

# Supplementary Table 1 Contact angle measurement and surface tension analysis

| Sample | Contact angle (°) | |  | Surface tension (mJ/m^2^) | | | | | |
| --- | --- | --- | --- | --- | --- | --- | --- | --- | --- |
|  | H_2_O | Diiodomethane |  | γ_s_^d^ | γ_s_^p^ | γ: STU/PIM-1 | γ: STU/PIM-1-300TR | γ: STU/PIM-1-350TR | γ: STU/PIM-1-400TR |
| MAF-STU-1 | 44.8 | 48.5 |  | 35.10 | 23.42 |  |  |  |  |
| PIM-1 | 93.4 | 38.6 |  | 40.30 | 0.41 | 17.78 |  |  |  |
| PIM-1-300TR | 109 | 24.1 |  | 46.46 | 1.03 |  | 15.39 |  |  |
| PIM-1-350TR | 112.8 | 13.2 |  | 49.47 | 2.17 |  |  | 12.54 |  |
| PIM-1-400TR | 70.5 | 59.1 |  | 29.09 | 10.70 |  |  |  | 2.74 |

# Supplementary Table 2 The diffusivity coefficient, solubility coefficient and their selectivities for different membranes at 0.5 bar.

| Membranes | D (×10^–6^ cm^2^/s) | | S (×10^–2^ cm^3^ (STP)/cm^3^·cmHg) | | α (CO_2_/N_2_) | |
| --- | --- | --- | --- | --- | --- | --- |
|  | CO_2_ | N_2_ | CO_2_ | N_2_ | D(CO_2_/N_2_) | S(CO_2_/N_2_) |
| PIM-1 (S0) | 10.22 | 11.08 | 5.99 | 0.31 | 0.92 | 19.50 |
| S8% | 35.56 | 20.91 | 3.17 | 0.22 | 1.70 | 14.43 |
| S8%-300TR | 37.51 | 15.65 | 3.20 | 0.26 | 2.40 | 12.21 |
| S8%-350TR | 43.70 | 6.38 | 2.90 | 0.37 | 6.85 | 7.49 |
| S40% | 18.75 | 10.64 | 4.66 | 0.43 | 1.76 | 10.77 |
| S40%-300TR | 23.57 | 5.80 | 5.10 | 0.46 | 4.06 | 11.06 |
| S40%-350TR | 23.90 | 5.81 | 5.54 | 0.46 | 4.11 | 11.99 |

# Supplementary Table 3 Sorption parameters of different membranes, obtained by the model fitting of the CO_2_ and N_2_ sorption isotherms.

| Membranes | Gas | Sorption parameters | |
| --- | --- | --- | --- |
|  |  | *C’_i_* cm^3^ (STP)/cm^3^ | *b_i_* (1/bar) |
| S0% | CO_2_ | 61.53 | 1.15 |
|  | N_2_ | 8.35 | 0.32 |
| S8% | CO_2_ | 52.35 | 1.81 |
|  | N_2_ | 15.83 | 0.14 |
| S8%-300TR | CO_2_ | 50.37 | 1.98 |
|  | N_2_ | 9.32 | 0.32 |
| S8%-350TR | CO_2_ | 40.92 | 2.32 |
|  | N_2_ | 19.12 | 0.23 |
| S40% | CO_2_ | 57.08 | 4.27 |
|  | N_2_ | 19.61 | 0.23 |
| S40%-300TR | CO_2_ | 58.21 | 4.32 |
|  | N_2_ | 18.82 | 0.24 |
| S40%-350TR | CO_2_ | 60.96 | 4.48 |
|  | N_2_ | 17.38 | 0.30 |

# Supplementary Table 4. Comparisons of TR-MMMs performances on CO_2_/N_2_ selectivity and CO_2_ permeability with previously reported interfacial modified MMMs.

| Polymers | Fillers | CO_2_ Permeability/(Barrer) | Selectivity | Modification methods | Refs |
| --- | --- | --- | --- | --- | --- |
| PIM-1 | MAF-stu-1  PIM-1  S8%  S40%  S8%-300TR  S8%-350TR  S8%-400TR  S40%-300TR  S40%-350TR  S40%-400TR | 6500  11726  9481  12222  11012  4216  9537  10125  2011 | 18  28.14  19.32  38.07  45.46  42.90  40.03  46.78  55.13 | Thermal rearrangement | This work |
| PIM-1 | UiO-66-CN | 22665 | 28.6 | Filler functionalization | ^[17]^ |
| Pebax | NH_2_-ZIF-8 | 163.8 | 62 | Filler functionalization | ^[18]^ |
| Pebax | CuBDC-ns@MoS_2_ | 123 | 18 | Filler functionalization | ^[19]^ |
| PIM-PI | PIM-PI-functionalized MOFs | 3827.3 | 24 | Filler functionalization | ^[20]^ |
| Speek | PEI@MIL-101(Cr) | 2490 | 80 | Filler functionalization | ^[21]^ |
| Matrimid-5218 | trifluoroacetic acid (TFA) modified MOF-808 | 30 | 29.5 | Filler functionalization | ^[22]^ |
| PIM-1 | MOF-801/ionic liquid | 9420 | 29 | Additive | ^[23]^ |
| 6FDA-durene | HKUST-IL | 1101.6 | 29.3 | Additive | ^[24]^ |
| Pebax | IL@ZIF-8 | 104.9 | 83.9 | Additive | ^[25]^ |
| 6FDA-durene | ZIF-67/IL | 1529.86 | 13.6 | Additive | ^[26]^ |
| TB-polyimide | ZIF-8@PD-PI | 1056 | 14.5 | Additive | ^[27]^ |
| PIM-1 | TSIL@NH_2_-MIL-101(Cr) | 2979 | 37 | Additive | ^[28]^ |
| Matrimid@5218 | IL@Cu-BTC | 120 | >100 | Additive | ^[29]^ |
| PDMS | Corona-MOF (UiO-66-AllyI-C) | 3569 | 9.7 | Cross-linking | ^[30]^ |
| PEG/PPG-  PDMS | UiO-66-NB | 585 | 53 | Cross-linking | ^[31]^ |
| 6FDADAM-  Br | MIL-101(Cr) | 1981 | 18.6 | Cross-linking | ^[32]^ |
| PIM-1 | UiO-66-NH_2_ | 12498 | 54.2 | Cross-linking | ^[33]^ |
| PEI | PSM-nZIF-7 | 245.9 | 1.34 | Cross-linking | ^[34]^ |
| ODPA-DAM | UiO-66-NH_2_@PI | 142 | 27 | Cross-linking | ^[35]^ |

# Supplementary Table 5. Process simulation results of different membranes: required membrane area, cost, and compressor power demand under varying CO_2_ recoveries, serving as the basis for cost and performance evaluation.

|  | Membrane Cost (USD/m^2^) | Recovery (%) | Membrane Area （m^2^） | | | Total Power (kW) |
| --- | --- | --- | --- | --- | --- | --- |
|  |  |  | 1st | 2nd | Total areas |  |
| PIM-1 | 50 | 50 | 51825 | 3581 | 55406 | 614.8 |
|  |  | 60 | 68820 | 4401 | 73221 | 704 |
|  |  | 70 | 99590 | 4481 | 104071 | 877.9 |
|  |  | 80 | 118539 | 5758 | 124297 | 937.2 |
|  |  | 90 | 178719 | 6095 | 184814 | 1248 |
| S8% | 50 | 50 | 22140 | 2384 | 24524 | 506.8 |
|  |  | 60 | 32780 | 2512 | 35292 | 580.6 |
|  |  | 70 | 43990 | 2960 | 46950 | 650.1 |
|  |  | 80 | 61020 | 3262 | 64282 | 750.1 |
|  |  | 90 | 88120 | 3647 | 91767 | 892 |
| S8%-300TR | 55 | 50 | 20459 | 2895 | 23354 | 481.1 |
|  |  | 60 | 27742 | 3474 | 31216 | 522.6 |
|  |  | 70 | 37360 | 3884 | 41244 | 608.1 |
|  |  | 80 | 52914 | 4232 | 57146 | 637.4 |
|  |  | 90 | 81080 | 4214 | 85294 | 752.4 |
| S8%-350TR | 55 | 50 | 23420 | 3142 | 26562 | 474.7 |
|  |  | 60 | 31844 | 3769 | 35613 | 513.7 |
|  |  | 70 | 43210 | 4396 | 47606 | 558.5 |
|  |  | 80 | 59830 | 5023 | 64853 | 648.9 |
|  |  | 90 | 88890 | 5651 | 94541 | 695 |
| S40% | 50 | 50 | 53092 | 2891 | 55983 | 649.7 |
|  |  | 60 | 69905 | 3493 | 73398 | 750.4 |
|  |  | 70 | 89022 | 4040 | 93062 | 854.6 |
|  |  | 80 | 116357 | 4633 | 120990 | 999.3 |
|  |  | 90 | 163580 | 5289 | 168869 | 1241 |
| S40%-300TR | 55 | 50 | 33470 | 4189 | 37659 | 480 |
|  |  | 60 | 45550 | 5008 | 50558 | 521.9 |
|  |  | 70 | 61832 | 5848 | 67680 | 570.5 |
|  |  | 80 | 85610 | 6674 | 92284 | 631.6 |
|  |  | 90 | 126843 | 7488 | 134331 | 722.3 |
| S40%-350TR | 55 | 50 | 30677 | 4906 | 35583 | 473 |
|  |  | 60 | 41689 | 5899 | 47588 | 511.1 |
|  |  | 70 | 56440 | 6834 | 63274 | 553.9 |
|  |  | 80 | 77897 | 7850 | 85747 | 605.8 |
|  |  | 90 | 116840 | 8009 | 124849 | 686 |

# Supplementary Table 6. Techno-economic analysis of membrane processes: operating cost, capital cost, annual CO_2_ captured, and capture cost at different recoveries.

|  | Recovery (%) | OPEX (USD/year) | Capital Cost – Equipment (USD) | Capital Cost – Membrane (USD) | Annual CO_2_ Captured (ton/year) | CO_2_ Capture Cost (USD/ton) |
| --- | --- | --- | --- | --- | --- | --- |
|  |  |  |  |  |  |  |
| PIM-1 | 50 | 491,840 | 518,012 | 2,770,300 | 7,766.50 | 110.49 |
|  | 60 | 563,200 | 563,189 | 3,661,050 | 9,535.00 | 108.56 |
|  | 70 | 702,320 | 624,404 | 5,203,550 | 10,812.70 | 125.58 |
|  | 80 | 749,760 | 648,295 | 6,214,850 | 12,447.10 | 129.26 |
|  | 90 | 998,400 | 693,482 | 9,240,700 | 14,311.60 | 148.56 |
| S8% | 50 | 405,440 | 444,195 | 1,226,200 | 7,852.80 | 75.32 |
|  | 60 | 464,480 | 495,119 | 1,764,600 | 9,421.60 | 76.01 |
|  | 70 | 520,080 | 541,211 | 2,347,500 | 11,056.30 | 76.14 |
|  | 80 | 600,080 | 604,750 | 3,214,100 | 12,563.60 | 81.62 |
|  | 90 | 713,600 | 690,068 | 4,588,350 | 13,974.40 | 93.14 |
| S8%-300TR | 50 | 384,880 | 425,932 | 1,284,470 | 7,846.10 | 73.33 |
|  | 60 | 418,080 | 455,282 | 1,716,880 | 9,417.10 | 70.09 |
|  | 70 | 486,480 | 513,561 | 2,268,420 | 10,987.70 | 70.48 |
|  | 80 | 509,920 | 532,913 | 3,143,030 | 12,585.80 | 74.05 |
|  | 90 | 601,920 | 606,176 | 4,691,170 | 14,167.30 | 84.13 |
| S8%-350TR | 50 | 379,760 | 421,339 | 1,460,910 | 7,858.40 | 75 |
|  | 60 | 410,960 | 449,050 | 1,958,715 | 9,426.20 | 72.05 |
|  | 70 | 446,800 | 480,095 | 2,618,330 | 10,999.30 | 72 |
|  | 80 | 519,120 | 540,429 | 3,566,915 | 12,568.50 | 77.7 |
|  | 90 | 556,000 | 570,122 | 5,199,755 | 14,144.40 | 84.74 |
| S40% | 50 | 519,760 | 540,950 | 2,799,150 | 7,726.10 | 115.43 |
|  | 60 | 600,320 | 604,936 | 3,669,900 | 9,421.30 | 114.26 |
|  | 70 | 683,680 | 668,084 | 4,653,100 | 10,861.00 | 117.52 |
|  | 80 | 799,440 | 751,342 | 6,049,500 | 12,437.20 | 125.18 |
|  | 90 | 992,800 | 880,863 | 8,443,450 | 14,247.20 | 142.58 |
| S40%-300TR | 50 | 384,000 | 425,144 | 2,071,245 | 7,848.50 | 84.35 |
|  | 60 | 417,520 | 454,793 | 2,780,690 | 9,421.30 | 82.57 |
|  | 70 | 456,400 | 488,276 | 3,722,400 | 11,000.40 | 84.12 |
|  | 80 | 505,280 | 529,106 | 5,075,620 | 12,562.20 | 89.92 |
|  | 90 | 577,840 | 587,392 | 7,388,205 | 14,082.10 | 104.12 |
| S40%-350TR | 50 | 378,400 | 420,115 | 1,957,065 | 7,849.90 | 81.93 |
|  | 60 | 408,880 | 447,223 | 2,617,340 | 9,421.30 | 79.63 |
|  | 70 | 443,120 | 476,944 | 3,480,070 | 11,000.30 | 80.35 |
|  | 80 | 484,640 | 512,029 | 4,716,085 | 12,562.20 | 84.93 |
|  | 90 | 548,800 | 564,378 | 6,866,695 | 14,163.80 | 97.18 |

References

[1] M. J. Abraham, T. Murtola, R. Schulz, S. Páll, J. C. Smith, B. Hess, E. Lindahl, “GROMACS: High performance molecular simulations through multi-level parallelism from laptops to supercomputers” *SoftwareX* **2015**, *1–2*, 19–25.

[2] M. A. Addicoat, N. Vankova, I. F. Akter, T. Heine, “Extension of the Universal Force Field to Metal–Organic Frameworks” *J Chem Theory Comput* **2014**, *10*, 880–891.

[3] A. K. Rappe, C. J. Casewit, K. S. Colwell, W. A. I. I. I. Goddard, W. M. Skiff, “UFF, a full periodic table force field for molecular mechanics and molecular dynamics simulations” *J Am Chem Soc* **1992**, *114*, 10024–10035.

[4] D. E. Coupry, M. A. Addicoat, T. Heine, “Extension of the Universal Force Field for Metal–Organic Frameworks” *J Chem Theory Comput* **2016**, *12*, 5215–5225.

[5] J. Wang, R. M. Wolf, J. W. Caldwell, P. A. Kollman, D. A. Case, “Development and testing of a general amber force field” *J Comput Chem* **2004**, *25*, 1157–1174.

[6] A. Gabrieli, M. Sant, P. Demontis, G. B. Suffritti, “Partial Charges in Periodic Systems: Improving Electrostatic Potential (ESP) Fitting via Total Dipole Fluctuations and Multiframe Approaches” *J Chem Theory Comput* **2015**, *11*, 3829–3843.

[7] G. Bussi, D. Donadio, M. Parrinello, “Canonical sampling through velocity rescaling” *J Chem Phys* **2007**, *126*, 014101.

[8] X. He, C. Fu, M.-B. Hägg, “Membrane system design and process feasibility analysis for CO2 capture from flue gas with a fixed-site-carrier membrane” *Chemical Engineering Journal* **2015**, *268*, 1–9.

[9] X. He, M.-B. Hägg, T.-J. Kim, “Hybrid FSC membrane for CO2 removal from natural gas: Experimental, process simulation, and economic feasibility analysis” *AIChE Journal* **2014**, *60*, 4174–4184.

[10] D. Grainger, M.-B. Hägg, “The recovery by carbon molecular sieve membranes of hydrogen transmitted in natural gas networks” *Int J Hydrogen Energy* **2008**, *33*, 2379–2388.

[11] Y. Chu, X. He, “Process simulation and cost evaluation of carbon membranes for CO 2 removal from high-pressure natural gas” *Membranes (Basel)* **2018**, *8*, DOI 10.3390/membranes8040118.

[12] X. He, Y. Chu, A. Lindbråthen, M. Hillestad, M.-B. Hägg, “Carbon molecular sieve membranes for biogas upgrading: Techno-economic feasibility analysis” *J Clean Prod* **2018**, *194*, 584–593.

[13] R. Turton, R. C. Bailie, W. B. Whiting, J. A. Shaeiwitz, D. Bhattacharyya, *Analysis, Synthesis, and Design of Chemical Processes Fourth Edition*, **n.d.**

[14] X. He, “Polyvinylamine-Based Facilitated Transport Membranes for Post-Combustion CO2 Capture: Challenges and Perspectives from Materials to Processes” *Engineering* **2021**, *7*, 124–131.

[15] X. He, L. Lei, Z. Dai, “Green hydrogen enrichment with carbon membrane processes: Techno-economic feasibility and sensitivity analysis” *Sep Purif Technol* **2021**, *276*, 119346.

[16] L. Lei, A. Lindbråthen, M. Hillestad, X. He, “Carbon molecular sieve membranes for hydrogen purification from a steam methane reforming process” *J Memb Sci* **2021**, *627*, 119241.

[17] G. Yu, X. Zou, L. Sun, B. Liu, Z. Wang, P. Zhang, G. Zhu, “Constructing Connected Paths between UiO-66 and PIM-1 to Improve Membrane CO2 Separation with Crystal-Like Gas Selectivity” *Advanced Materials* **2019**, *31*, 1806853.

[18] R. Ding, W. Zheng, K. Yang, Y. Dai, X. Ruan, X. Yan, G. He, “Amino-functional ZIF-8 nanocrystals by microemulsion based mixed linker strategy and the enhanced CO2/N2 separation” *Sep Purif Technol* **2020**, *236*, DOI 10.1016/j.seppur.2019.116209.

[19] N. Liu, J. Cheng, W. Hou, C. Yang, X. Yang, J. Zhou, “Bottom-up synthesis of two-dimensional composite via CuBDC-ns growth on multilayered MoS2 to boost CO2 permeability and selectivity in Pebax-based mixed matrix membranes” *Sep Purif Technol* **2022**, *282*, 120007.

[20] M. Mubashir, Y. F. Yeong, K. K. Lau, T. L. Chew, J. Norwahyu, “Efficient CO2/N2 and CO2/CH4 separation using NH2-MIL-53(Al)/cellulose acetate (CA) mixed matrix membranes” *Sep Purif Technol* **2018**, *199*, 140–151.

[21] Q. Xin, J. Ouyang, T. Liu, Z. Li, Z. Li, Y. Liu, S. Wang, H. Wu, Z. Jiang, X. Cao, “Enhanced Interfacial Interaction and CO2 Separation Performance of Mixed Matrix Membrane by Incorporating Polyethylenimine-Decorated Metal–Organic Frameworks” *ACS Appl Mater Interfaces* **2015**, *7*, 1065–1077.

[22] R. Thür, D. Van Havere, N. Van Velthoven, S. Smolders, A. Lamaire, J. Wieme, V. Van Speybroeck, D. De Vos, I. F. J. Vankelecom, “Correlating MOF-808 parameters with mixed-matrix membrane (MMM) CO2permeation for a more rational MMM development” *J Mater Chem A Mater* **2021**, *9*, 12782–12796.

[23] W. Chen, Z. Zhang, C. Yang, J. Liu, H. Shen, K. Yang, Z. Wang, “PIM-based mixed-matrix membranes containing MOF-801/ionic liquid nanocomposites for enhanced CO2 separation performance” *J Memb Sci* **2021**, *636*, DOI 10.1016/j.memsci.2021.119581.

[24] R. Lin, L. Ge, H. Diao, V. Rudolph, Z. Zhu, “Ionic liquids as the MOFs/polymer interfacial binder for efficient membrane separation” *ACS Appl Mater Interfaces* **2016**, *8*, 32041–32049.

[25] H. Li, L. Tuo, K. Yang, H. K. Jeong, Y. Dai, G. He, W. Zhao, “Simultaneous enhancement of mechanical properties and CO2 selectivity of ZIF-8 mixed matrix membranes: Interfacial toughening effect of ionic liquid” *J Memb Sci* **2016**, *511*, 130–142.

[26] M. T. Vu, R. Lin, H. Diao, Z. Zhu, S. K. Bhatia, S. Smart, “Effect of ionic liquids (ILs) on MOFs/polymer interfacial enhancement in mixed matrix membranes” *J Memb Sci* **2019**, *587*, DOI 10.1016/j.memsci.2019.05.081.

[27] Z. Wang, D. Wang, S. Zhang, L. Hu, J. Jin, “Interfacial Design of Mixed Matrix Membranes for Improved Gas Separation Performance” *Advanced Materials* **2016**, *28*, 3399–3405.

[28] J. Ma, Y. Ying, X. Guo, H. Huang, D. Liu, C. Zhong, “Fabrication of mixed-matrix membrane containing metal-organic framework composite with task-specific ionic liquid for efficient CO2 separation” *J Mater Chem A Mater* **2016**, *4*, 7281–7288.

[29] B. Monteiro, A. R. Nabais, M. H. Casimiro, A. P. S. Martins, R. O. Francisco, L. A. Neves, C. C. L. Pereira, “Impact on CO2/N2 and CO2/CH4 separation performance using Cu-BTC with supported ionic liquids-based mixed matrix membranes” *Membranes (Basel)* **2018**, *8*, DOI 10.3390/membranes8040093.

[30] Y. Katayama, K. C. Bentz, S. M. Cohen, *Defect-Free MOF-Based Mixed Matrix Membranes Obtained from Corona Crosslinking*, **n.d.**

[31] I. Hossain, A. Husna, S. Chaemchuen, F. Verpoort, T. H. Kim, “Cross-Linked Mixed-Matrix Membranes Using Functionalized UiO-66-NH2into PEG/PPG-PDMS-Based Rubbery Polymer for Efficient CO2Separation” *ACS Appl Mater Interfaces* **2020**, *12*, 57916–57931.

[32] J. H. Jo, K. J. Kim, E. J. An, J. Lee, H. Jae, D. Roh, W. S. Chi, “Ionic Cross-Linked MOF-Polymer Mixed-Matrix Membranes for Suppressing Interfacial Defects and Plasticization Behavior” *ACS Appl Mater Interfaces* **2023**, DOI 10.1021/acsami.3c19071.

[33] N. Tien-Binh, D. Rodrigue, S. Kaliaguine, “In-situ cross interface linking of PIM-1 polymer and UiO-66-NH2 for outstanding gas separation and physical aging control” *J Memb Sci* **2018**, *548*, 429–438.

[34] B. A. Al-Maythalony, A. M. Alloush, M. Faizan, H. Dafallah, M. A. A. Elgzoly, A. A. A. Seliman, A. Al-Ahmed, Z. H. Yamani, M. A. M. Habib, K. E. Cordova, O. M. Yaghi, “Tuning the interplay between selectivity and permeability of ZIF-7 mixed matrix membranes” *ACS Appl Mater Interfaces* **2017**, *9*, 33401–33407.

[35] H. Wang, S. He, X. Qin, C. Li, T. Li, “Interfacial Engineering in Metal-Organic Framework-Based Mixed Matrix Membranes Using Covalently Grafted Polyimide Brushes” *J Am Chem Soc* **2018**, *140*, 17203–17210.
